# Pre- and Postharvest Strategies for *Pleurotus ostreatus* Mushroom in a Circular Economy Approach

**DOI:** 10.3390/foods13101464

**Published:** 2024-05-09

**Authors:** Mafalda Silva, Ana Cristina Ramos, Fernando J. Lidon, Fernando H. Reboredo, Elsa M. Gonçalves

**Affiliations:** 1 INIAV—Instituto Nacional de Investigação Agrária e Veterinária, Unidade de Tecnologia e Inovação, 2780-157 Oeiras, Portugal; mafaldasdos@gmail.com (M.S.);; 2 Faculdade de Ciências e Tecnologia (FCT), Universidade NOVA de Lisboa (UNL), 1600-560 Caparica, Portugal; 3 GeoBioTec—Geobiociências, Geoengenharias e Geotecnologias, Faculdade de Ciências e Tecnologia, Universidade NOVA de Lisboa, 2829-516 Caparica, Portugal

**Keywords:** edible mushrooms, *Pleurotus ostreatus*, valorization of residues

## Abstract

Mushroom cultivation presents a viable solution for utilizing agro-industrial byproducts as substrates for growth. This process enables the transformation of low-economic-value waste into nutritional foods. Enhancing the yield and quality of preharvest edible mushrooms, along with effectively preserving postharvest mushrooms, stands as a significant challenge in advancing the industry. Implementing pre- and postharvest strategies for *Pleurotus ostreatus* (Jacq.) P. Kumm (oyster mushroom) within a circular economy framework involves optimizing resource use, minimizing waste, and creating a sustainable and environmentally friendly production system. This review aimed to analyze the development and innovation of the different themes and trends by bibliometric analysis with a critical literature review. Furthermore, this review outlines the cultivation techniques for *Pleurotus ostreatus*, encompassing preharvest steps such as spawn production, substrate preparation, and the entire mushroom growth process, which includes substrate colonization, fruiting, harvesting, and, finally, the postharvest. While novel methodologies are being explored for maintaining quality and extending shelf-life, the evaluation of the environmental impact of the entire mushroom production to identify areas for improvement is needed. By integrating this knowledge, strategies can be developed for a more sustainable and circular approach to *Pleurotus ostreatus* mushroom cultivation, promoting environmental stewardship and long-term viability in this industry.

## 1. Introduction

Along with world population growth, there is an increased demand for the supply of food products, generating pressure on the agro-industry sector and consequently on the volume of residues, which will inevitably increase [1]. Approximately 16 million tons of byproducts are output by the European agro-industry [2]. According to Sharmin et al. [3], these byproducts are likely to have a negative impact on water and air quality due to the release of gases such as carbon dioxide, methane, and nitric oxide, raising concerns for human health and the environment. Agricultural and food industry byproducts rich in phytochemicals, proteins, and polysaccharides offer valuable opportunities for commercial utilization and waste reduction, thus aligning with the European Commission’s emphasis on a circular economy [4]. This approach is expected to create value while managing byproducts in a sustainable and eco-friendly manner. The overarching goal is likely to promote environmental sustainability, reduce waste, and contribute to mitigating climate change.

The mushroom industry is based on the production of edible, medicinal, and wild mushrooms, the first being the main ones on the market. Over the years, there has been an increase in mushroom production worldwide, with China remaining in the lead. Poland is the main mushroom-producing country at the European level, followed by the Netherlands [5]. Mushroom production in Portugal reflects a dynamic industry that continues to adapt to the demands of both the domestic and export markets and to sustainable practices that have gained a lot of attention from different actors in the chain.

Mushroom cultivation has demonstrated both its economic viability and ecological significance by efficiently utilizing, adding value to, and bio-transforming agro-industrial residues. The current literature indicates that some mushroom species can also be utilized in various other applications, including the bioremediation and biodegradation of hazardous compounds. This approach contributes to the circular economy by transforming low-economic-value waste into high-nutritional-value food. However, despite their similar morphologies, mushroom species require different growth conditions [6].

Belonging to the *Pleurotus* genus, oyster mushrooms (*Pleurotus* spp.) make up an important proportion of the edible mushrooms cultivated worldwide. They are renowned for their distinct appearance, delightful taste, and nutritional benefits. They may present different shades of white, cream, gray, yellow, pink, or light brown in an oyster-shaped form [7]. Their ability to absorb flavors makes them versatile in cooking. They are used in various recipes, such as salads, stir-fries, soups, and vegetarian dishes. *Pleurotus ostreatus* (Jacq.) P. Kumm. is one of the main edible mushroom species produced worldwide [5] due to its ease of adaptation to the edaphoclimatic conditions of the environment and the ability to degrade a wide spectrum of substrates [8]. To produce this species, wheat straw is used as a very common substrate [9]; however, several studies use a combination of other agro-industrial byproducts [10,11].

The nutritional and functional aspects of mushrooms are exceptional. Mushrooms are low in calories and fats and rich in proteins (16.5–37.0%) and dietary fiber (24.4–46.6%), as well as minerals, such as phosphorus, potassium, iron, copper, and zinc [12]. All essential amino acids are present in mushrooms, and polyunsaturated fatty acids, particularly oleic and linoleic, are present in higher proportions than saturated fatty acids [13]. Numerous studies have shown the functional benefits of edible mushrooms in preventing some chronic conditions [13,14]. Because of these numerous benefits, there are now many ways to preserve the quality of mushrooms in general to increase their shelf-life. These approaches include applying both conventional and novel preservation techniques [15,16]. Balancing the benefits of extended shelf-life with maintaining the sensory and nutritional qualities of mushrooms is an ongoing challenge in the food industry. The choice of preservation method depends on factors such as the intended use of the mushrooms and consumer preferences. Mushroom byproduct valorization involves finding creative and sustainable ways to utilize these byproducts for economic, environmental, or social benefits. Mushroom byproduct valorization refers to the process of extracting value from the waste or byproducts generated during the cultivation and processing of mushrooms. In addition to being a well-liked and wholesome food source, mushrooms produce a variety of byproducts from their cultivation that can be used again instead of being thrown away. In this regard, some potential avenues for mushroom byproduct valorization have been presented and discussed, for example, compost and soil enrichment [17], enrichment techniques for other foods [18,19], bioactive compound extraction [20], mushroom-based packaging materials [21,22], and soil bioremediation [23].

In this review, numerous facets of the physiology of *Pleurotus* sp. are highlighted, along with the effects of various nutritional and environmental factors on the growth of the mycelium and the creation of the basidioma. The cultivation techniques for *Pleurotus ostreatus* species are also described. These techniques encompass spawn production (inoculum), substrate preparation, and the cultivation of mushrooms, involving various stages. These stages comprise inoculation, the colonization of the substrate by the cultivated fungus, the fruiting phase, harvesting, and the processing of the resulting basidioma. Acknowledging the necessity of evaluating mushroom production and postharvest processes is crucial. Therefore, this review aims to conduct a life-cycle assessment to allow a comprehensive understanding of the different strategies to underscore the holistic approach needed for sustainable mushroom cultivation and help to identify areas for improvement and optimization.

### 1.1. Bibliometric Study

**Figure 1:**
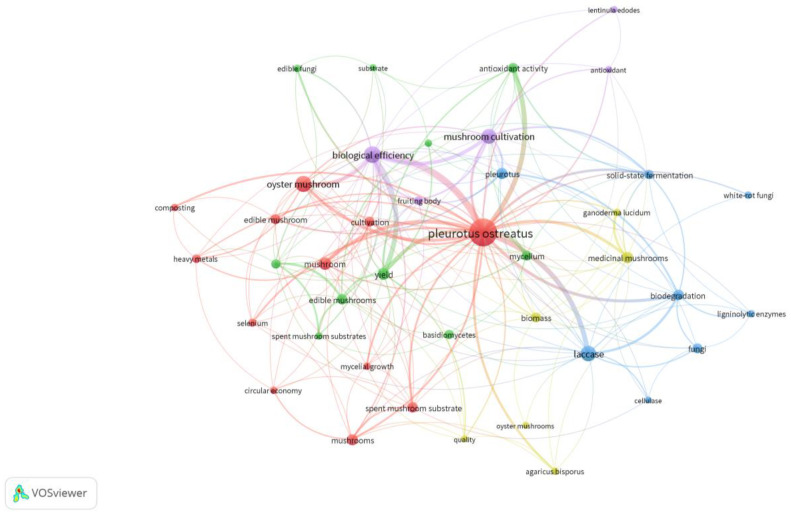
An overview of the important terms associated with *Pleurotus ostreatus* cultivation and postharvest practices.

To illustrate the knowledge network surrounding the theme of *Pleurotus ostreatus*, specifically focusing on cultivation methods and postharvest practices, a bibliometric analysis was performed using VOSviewer software v 1.6.19. This software makes it possible to create maps of authors or journals based on co-citation data or to construct maps of keywords based on co-occurrence data [24]. The creation of a term map to visualize the co-occurrence of key terms, as depicted in Figure 1, offers valuable insights into the thematic landscape of the research. Through co-occurrence network analysis, it becomes evident that certain terms are recurrent and central to the discourse. 

For the research strategy, the database used was Web of Science Core Collection, using the following keywords [“*Pleurotus ostreatus*” AND (“postharvest” OR “cultivation”)]. The results of the search identified 814 publications on the subject in the time span from 1968 to 2023. To refine the results, only articles from 2013 to 2023 were selected, after which the number of publications remaining dropped to 479, which were further analyzed using the software mentioned above.

A term map was created to visualize the co-occurrence of key terms and is displayed in Figure 1. The co-occurrence network analysis of the papers showed the five most common author keywords (co-occurrence frequency >5 times): “*Pleurotus ostreatus*”, “biological efficiency”, “yield”, “laccase”, and “mushroom cultivation”. Author keywords consist of terms that authors consider to best describe their work [25].

The first notable keyword is “*Pleurotus ostreatus*”, which likely signifies the focus on this particular species within the context of the research. *P. ostreatus*, commonly known as the oyster mushroom, holds significance in various fields, including biotechnology and agriculture, owing to its versatile properties and potential applications. Following closely are keywords such as “biological efficiency” and “yield”, indicating a strong emphasis on the productivity and effectiveness of mushroom cultivation processes. These terms underscore the practical implications of the research, suggesting a keen interest in optimizing production outcomes and resource utilization. The inclusion of “laccase” among the top keywords is noteworthy, highlighting the relevance of enzymatic processes in mushroom cultivation. Laccase, an enzyme with diverse functions, plays a crucial role in various biotechnological applications, including the degradation of lignocellulosic materials and the enhancement of fungal growth. Moreover, the term “mushroom cultivation” itself emerges as a significant keyword, reaffirming the overarching theme of the research. This indicates a comprehensive exploration of cultivation techniques, environmental factors, and biotechnological interventions aimed at improving mushroom yield and quality.

The visualization of keyword co-occurrences, with the node size representing frequency and the line thickness indicating co-occurrence frequency, offers a nuanced understanding of the interrelations between different concepts within the research domain. Such analyses not only facilitate knowledge synthesis but also inform future research directions by identifying key areas of interest and potential avenues for exploration.

Therefore, the term map and co-occurrence network analysis provide a structured framework for comprehending the central themes and connections within the researched literature. By highlighting key terms such as *Pleurotus ostreatus*, biological efficiency, yield, laccase, and mushroom cultivation, this analysis sheds light on the multifaceted aspects of mushroom research and underscores the importance of interdisciplinary approaches in addressing contemporary challenges in agriculture and biotechnology.

**Figure 2:**
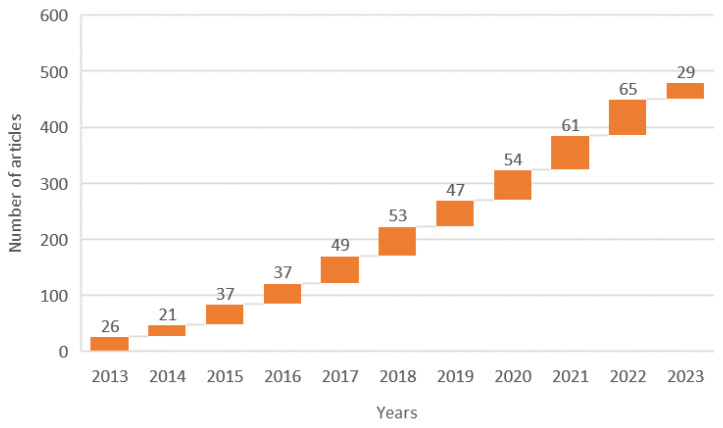
The publication trend in the cultivation and postharvest topic for Pleurotus ostreatus.

Figure 2 portrays the publication trend of articles that were published between 2013 and 2023 about the cultivation and postharvest processing of *Pleurotus ostreatus* (data exported from the Web of Science database). About 96% of these publications were written in English; the remaining were in Spanish, Russian, and Indonesian. China was the largest contributor of publications, contributing 24.4% of 479 articles.

The literature surveyed highlights a noticeable gap in comprehensive reviews addressing postharvest strategies in the cultivation and utilization of mushrooms, particularly those centered on health-conscious and environmentally friendly preservation methods. Despite the upward trajectory observed in this field in recent years, a mere 7% of publications from the period 2013–2023 constituted review articles focusing on cultivation technologies and mushroom waste valorization. Unfortunately, none of these reviews thoroughly explore the discussion regarding postharvest strategies aimed at implementing more health-conscious and natural solutions to enhance mushroom preservation, mitigate environmental impacts, and contribute significantly to the sustainability of the mushroom supply chain.

In the upcoming sections, this review further explores the distinctive features of *Pleurotus ostreatus*. Understanding the unique features of this mushroom variety is essential for gaining insights into its cultivation, postharvest management, and utilization.

### 1.2. Pleurotus ostreatus Characteristics

**Figure 3:**
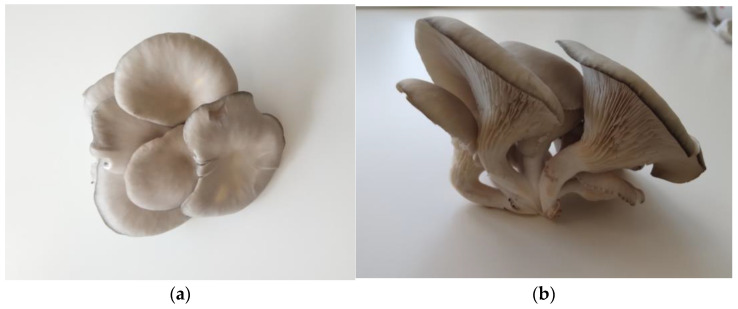
*Pleurotus ostreatus* morphology: (**a**) oyster-shaped gray cap, viewed from above; (**b**) gills and stipe, viewed from below.

*Pleurotus ostreatus*, commonly known as the oyster mushroom, is a popular edible mushroom with distinct morphological characteristics, as seen in Figure 3. Table 1 presents its taxonomic description.

The oyster mushroom comprises three distinctive components: (1) an oyster-shaped cap known as the pileus, as illustrated in Figure 3a; (2) a short or long stalk (stipe) located laterally or centrally; and (3) extended ridges and furrows beneath the pileus referred to as gills, as seen in Figure 3b. The cap diameter can vary from 5 to 11 cm diameter; the stipe length ranges from 4.7 to 7.2 cm, and its diameter from 1.3 to 2.2 cm [26].

The mushroom’s basidioma displays various hues, such as white, gray, yellow, pink, or light brown, and these colors are influenced by the specific species, such as *Pleurotus citrinopileatus*, which presents a vibrant yellowish color [27]. Additionally, shades may vary based on environmental conditions.

*Pleurotus ostreatus* possesses a diverse range of biochemical compounds. Key components in this regard include polysaccharides such as β-glucans, which are renowned for their contribution to the major pharmaceutical properties of mushrooms—specifically antitumor activities and immunity potentiation [28]. In this species, there is a specific β-glucan known as Pleuran. It has demonstrated a notable reduction in the occurrence of symptoms related to upper respiratory tract infections [29]. Additionally, phenolic compounds, predominantly hydroxybenzoic acid, and terpenoids like sterols, with ergosterol as the primary representative, contribute to the mushroom’s biochemical profile and antioxidant properties. Notably, ergosterol plays a crucial role in the fungal cell membrane. When exposed to UV irradiation, it undergoes a transformation into vitamin D_2_, presenting potential health benefits [30]. Further exploration of the health benefits of mushrooms is crucial. Future studies should develop formulations for incorporating these active metabolites into dietary supplements. This would involve identifying and isolating specific compounds, optimizing extraction methods, and exploring delivery mechanisms to ensure bioavailability. Additionally, research could delve into the synergistic effects of combining different mushroom species for enhanced health benefits. It is important to note that while the potential health benefits of mushrooms are promising, more research is needed to understand the mechanisms of action, the optimal dosage, and long-term effects [31].

In addition to its morphological and biochemical distinctiveness, *Pleurotus ostreatus* exhibits noteworthy physiological characteristics. As a saprophytic organism, it derives nutrients by decomposing organic matter [32]. A detailed examination of several physiological factors, including substrate utilization, pH preferences, and temperature needs, is provided in the next portion of this review. Understanding these key physiological traits is essential for optimizing cultivation strategies.

## 2. Mushroom Preharvest Activities

**Figure 4:**
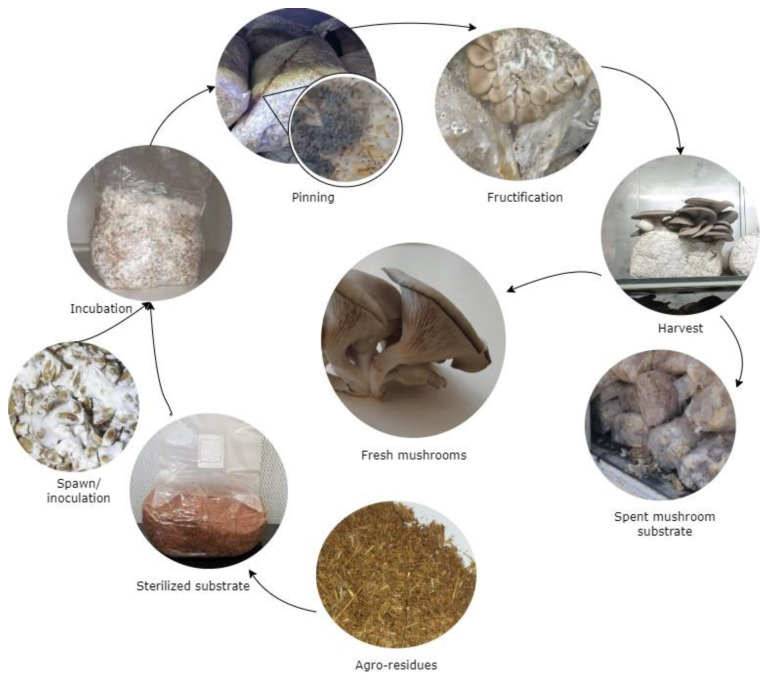
Preharvest activities in mushroom cultivation.

The cultivation of mushrooms is the practice of growing mushrooms for human consumption or various other purposes. It is essential to recognize what cultivation methods are specific to the mushroom species and the system employed. Preharvest activities in mushroom cultivation encompass several stages, commencing with spawn preparation, proceeding to substrate inoculation, advancing through spawn running, and culminating in the fructification of mushrooms [33]. Figure 4 provides a visual representation of the sequential stages involved in mushroom cultivation, starting from spawn preparation and culminating in the harvesting of fresh mushrooms.

Throughout the different phases, several requirements must be fulfilled. Regarding substrates, although moisture, pH, temperature, and the C:N ratio are the main parameters for the growth of mushrooms, other nutrients, such as phosphorus, magnesium, sulfur, calcium, iron, and potassium, as well as vitamins, should be monitored and possibly added to the growing media to enhance the biological efficiency of *Pleurotus ostreatus* [34]. Moreover, other factors, such as the particle size of the substrate materials, the amount of spawn used, mineral content, and luminosity, also have an impact on cultivation [35].

Additionally, maintaining cleanliness throughout the process is crucial in preventing contamination and ensuring a successful harvest. A basic overview of the steps involved in mushroom cultivation before harvest is presented in the following subsections.

### 2.1. Spawn

The spawn, which constitutes the pure culture of the vegetative mycelium within a propagation medium, functions as the primary inoculum for mushroom cultivation [36], serving as the mushroom analog to the seed in crop plants. Due to the complexity and resource-intensive nature of spawn cultivation, encompassing equipment, facilities, and labor, mushroom producers typically opt to procure it from specialized companies.

Spawn can be marketed as “solid spawn” or “liquid spawn”. As a propagation medium, solid spawn uses grains, e.g., wheat, rye, and oats, or lignocellulosic materials, such as sawdust and cottonseed hulls, as well as stick spawn [33]. Most studies use grain spawn because it is quick and easy to handle. To cultivate this type of spawn, grains undergo thorough washing to eliminate any floating particles, followed by boiling to facilitate water absorption. Subsequently, they are dried to remove excess moisture and mixed with carbonates to reduce stickiness and regulate pH levels. The treated grains are then transferred into containers and subjected to sterilization in an autoclave, preparing them for subsequent inoculation with a pure culture [37,38,39]. 

Each medium possesses distinct characteristics that significantly influence the process of mushroom cultivation. For instance, for solid spawn, sawdust is a widely used propagation medium because of its ease of use and low-cost production [40]. However, in comparison with sawdust, the use of stick spawn increases mushroom yields [33]. A drawback associated with stick spawn usage is the requirement for wood from trees. In this regard, Lui et al. [41] experimented with corn stalks as an alternative for spawn production. In their study, it was found that, after immersion in liquid culture, the mycelium of *P. ostreatus* adhered to the rough surface of the stalk. When used as the spawn in bag cultivation, the mycelium–stalk showed a very similar colonization period, approximately 12 days, but achieved a higher biological efficiency (69.5%), approximately 2% more than that of stick spawn. Regarding grain spawn, the advantage of using small grains as the spawn is that they allow a greater number of inoculation spots, and thus, the mycelium covers the substrate faster [36]; however, grain spawn presents a high contamination rate, and it is an expensive option [42]. According to Gupta et al. [43], the contamination rate varies depending on the grain type. In their study, sorghum presented a lower infection percentage (12.7%), while pearl millet had a significantly higher infection rate (20.7%). 

According to Zhang et al. [42], liquid spawn offers several advantages compared with solid spawn in terms of the low cost of production and space requirements; it is spread evenly on the substrate and allows automatic inoculation and a faster spawn running time. Ma et al. [40] also indicate that liquid spawn produces a more vigorous mycelium with less chance of contamination once it is produced under more aseptic conditions. It is produced by submerged fermentation, where nutrient sources affect the production of the mycelium [44]. Different carbon and nitrogen sources were tested by Ma et al. to evaluate the effect of the fermentation medium on mycelial biomass. Previous data indicate that combined sources of carbon stimulate fermentation, so in their study, the liquid spawn formulation was optimized for maximum mycelium biomass: 10% glucose, 3% corn flour, 3% glutinous rice flour, 0.2% fish peptone, and 0.3% KH_2_PO_4_ [40]. To inoculate liquid spawn, Zhang et al. [42] tested different inoculum materials, such as corncob, loofah sponge, sugarcane bagasse, and synthetic polyurethane foam. Although mycelia adhered to all supports, the mycelia of *P. ostreatus* exhibited the lowest metabolic activity on synthetic polyurethane and the highest on corncob, which can be an alternative medium for mushroom cultivation.

### 2.2. Substrate Preparation

Implementing a circular economy model involves recycling and assigning value to materials that were previously considered waste. Mushrooms play a crucial role in this transition due to their capability to convert organic matter into valuable products [45]. Cellulose, the most abundant polysaccharide found in agricultural and industrial fruit and vegetable wastes, serves as a prime example [46]. Mushrooms possess enzymes such as laccase, Mn-peroxidase, and ligninase, along with hydrolytic enzymes like cellulase, xylanase, and tannase, which facilitate the degradation of cellulose and lignin. These enzymes break down the substrate into soluble, low-molecular-weight compounds, making them accessible for further utilization [35,47].

However, choosing an appropriate substrate for mushroom production to obtain the appropriate physical, chemical, and biological characteristics, maximize production, and improve product quality can be a challenge.

Usually, to produce *P. ostreatus*, wheat straw is commonly used as a substrate. Masevhe et al. [48] said that it improves mycelium colonization and prevents *Trichoderma* spp. contamination. Most of the formulations referred to in Table 2 have wheat straw as a substrate base (mainly providing carbon), although, for the growth of mushrooms, it turns out to be necessary to add a source of nitrogen to maintain a favorable C:N ratio for growth at different stages [49], besides potassium and phosphorus.

Through supplementation, the ratio can be balanced, leading to different outputs. A variety of agro-industrial residues have been studied as supplements or alternative growth substrates, particularly molasses, brewer’s grain, paper waste, cotton, coffee wastes, and grain milling byproducts [50]. These residues, as referred to in Table 2, are the most commonly used supplements. This variation shows that mycelial growth efficiency and mushrooms’ physical and nutritional characteristics are majorly affected by the substrate formulation [51,52].

**Table 2:** Different substrate formulations for *Pleurotus ostreatus* production based on agro-industrial residues.

Substrates	Reference
Sawdust, cotton seed, wheat straw, and paper waste	[53]
Tea residues	[54]
Vegetable waste and rice straw	[49]
Wheat straw, spent ground coffee, and cardboard	[55]
Defatted almond meal, chicken manure, and wheat straw	[56]
Olive pomace and wheat straw	[57]
Wheat straw and spent ground coffee	[58]
Wheat straw, spent ground coffee, and olive pruning residues	[9]
Rice straw, wheat straw, corncobs, sawdust and rice husk, and sugarcane bagasse	[10]
Light coarse fiber residues, Betula spp., sawdust, wheat bran	[59]
Coffee pulp and wheat straw	[60]
Spent ground coffee and sawdust	[61]
Alfalfa pulp	[11]
Spent brewery grains, wheat bran, and beech sawdust	[62]
Palm waste, rice bran, and wheat bran	[63]

Substrates formulated with different residues exhibited impacts on incubation time, productivity, and the nutritional content of the final product.

Akter et al. [10] observed that a substrate comprising sawdust and rice husk resulted in a superior yield and higher concentrations of polyphenols. Similarly, Zhou et al. [11] demonstrated that employing alfalfa pulp as an alternative substrate yielded promising outcomes in terms of biological efficiency and amino acid composition when compared to the use of wheat straw. The basidioma that contained the highest amount of protein might be due to the availability of higher levels of nitrogen in the substrate where it grew [10,56]. Yolande et al. [64] noted that the amino acid composition of residues plays a crucial role in influencing the functional properties of mushrooms. Furthermore, the presence of the amino acid methionine in substrates formulated with corncobs and rice hulls was found to enhance the production of *P. ostreatus* lovastatin, a metabolite known for its hypocholesterolemic effects. Given mushrooms’ capacity to absorb and accumulate diverse mineral elements from their substrates, the mineral composition significantly influences the mineral content of mushrooms, including potentially harmful substances. Notably, mushrooms tend to concentrate metals primarily in their upper parts, often surpassing soil concentrations [65,66]. Golian et al. [66] assessed the mineral content of *P. ostreatus*, revealing an average range of concentrations from the highest at 30,000 mg/kg for potassium (K) to the lowest at 4.4 mg/kg for barium (Ba), with the order of abundance being K > Mg > Ca > Na > Zn > Fe > Cu > Al > Mn > Ba. In a separate investigation, Jin et al. [67] reported a slightly different element sequence, with the order being K > Mg > Na > Ca > Fe > Zn > Cu > Mn. Despite the variations, both studies consistently found potassium (K) and magnesium (Mg) to be the most abundant macroelements.

Biofortification involves enhancing the nutrient content of foods grown or processed in enriched media, offering a practical solution to combat micronutrient deficiencies in the diet [68]. Iron (Fe), zinc (Zn), manganese (Mn), and copper (Cu) are vital components of essential enzyme complexes in the human body, making mushrooms promising potential sources of these micronutrients [68,69]. Rzymski et al. [69] conducted an assay to explore substrate supplementation with Selenium (Se) alone or combined with Cu and/or Zn to enhance the mushroom’s nutritional value. Their findings indicate that Se supplementation led to biofortified basidioma containing 342–469% of the Recommended Daily Allowance (RDA) for Se, 43.4–48.5% for Cu, and 5.2–5.8% for Zn, with potential applications in nutrition and medicine.

When considering the substrate formulation for health reasons and improved biological efficiency, it is essential to recognize that the composition and treatment of the substrate influence the morphological characteristics of the mushroom [51,68].

#### 2.2.1. C:N Ratio

The C:N ratio stands out as a key parameter for mushrooms, exerting an influence on mycelium development during incubation, as well as mushroom growth in the fruiting phase, and on enzyme activity, which plays a crucial role in decomposing lignin-rich materials present in the substrate [70].

Agricultural residues, frequently serving as lignin-based substrates, as exemplified in Table 2, contribute significantly as carbon sources. It is well established that both carbon and nitrogen sources exert an influence on mushroom production [71].

According to Krupodorova et al. [72], various carbon sources support mycelial growth, such as glucose, fructose, xylose, starch, maltose, sucrose, mannose, and cellulose, among others. Reddy et al. [71] reported good growth in mineral salt broth containing mannitol as the carbon source and yeast extract as the nitrogen source.

Preferred nitrogenous sources for *Pleurotus* spp. include amino acids such as asparagine, alanine, glycine, arginine, and tryptophan and other complex organic compounds, mainly wheat bran, yeast extract, corn steep powder, and soybean cake powder [72].

For *Pleurotus ostreatus*, Laursen’s [73] findings demonstrate a preference for organic nitrogen sources over mineral nitrogen, such as nitrate. It exhibited superior growth outcomes when cultivated in a medium containing the amino acids L-glutamate and L-aspartate as nitrogen sources, alongside ammonium and yeast extract. 

Nonetheless, the predominant focus of ongoing studies concerning the C:N ratio in *Pleurotus ostreatus* cultivation lies in the comparison of various raw materials. This emphasis is driven by the objective of utilizing available waste materials inherent to specific regions or localities worldwide for mushroom production [72,74]. Therefore, managing the balance and quality of carbon and nitrogen sources in the substrate is vital for achieving optimal mushroom cultivation outcomes.

According to Chang and Miles [75], *P. ostreatus* demonstrates the ability to metabolize substantial amounts of carbon, including lignin, with minimal nitrogen presence. The C:N ratio for the genus *Pleurotus* is typically in the range of 45–60:1 [34]. Cueva et al. [76] reported higher biological efficiency for a 38–58:1 C:N ratio, with a maximum peak at a 38–48:1 C:N ratio. According to Yang et al. [51], higher ratios favor mycelial growth, while lower ratios are conducive to basidioma development. In Hoa et al. [77], it is noted that the composition of different substrates affects the colonization period. For instance, a substrate comprising 80% cotton seed hull (C:N = 34.9) required a longer time for full colonization compared to substrates formulated with 80% rice straw (C:N = 49.2) or 80% wheat straw (C:N = 64.6).

An imbalanced ratio can hinder mycelial growth, leading to reduced yields and biological efficiency [10], while potentially encouraging pathogenic microorganisms. Elevated nitrogen levels may also foster the presence of competitive threats such as *Trichoderma* [78].

However, the C:N ratio undergoes changes throughout the production cycle. As carbonic compounds break down, the C:N ratio decreases, resulting in increased nitrogen levels [78]. This aligns with the understanding that, during basidioma development, a lower C:N ratio in the cultivation substrate is more favorable [35].

#### 2.2.2. pH

The pH is a crucial factor to consider when preparing the substrate, as different raw materials can influence this aspect. As detailed in Hoa et al. [77], incorporating corncobs and sugarcane bagasse into substrate formulations led to a decrease in both the C:N ratio and pH value in comparison to substrates composed solely of sawdust.

For the fruiting phase, the optimal pH is between 4.0 and 7.0. However, as colonization occurs, the pH decreases, so the initial adjustment should be made to a pH between 6.5 and 7.0 through the use of a corrective, such as Ca_2_CO_3_ [35].

In an assay [79] where pH levels ranged from 5.0 to 6.4, the duration from pinhead emergence to the first harvest varied significantly. The shortest duration was observed at pH = 5.0. However, while the number of mushrooms produced gradually decreased with increasing pH from 5.0 to 5.8, a peak was noted at pH 6.1, demonstrating the maximum yield. Therefore, the pH value influences the proper growth and development of mushrooms. 

#### 2.2.3. Moisture

According to Chang and Miles [75], the water content of the substrate should be between 50% and 75% for optimal growth.

Elevated substrate moisture reduces porosity, limits oxygen, causes respiratory challenges for the mycelium, inhibits perspiration, and hampers basidioma development, potentially fostering the growth of pathogenic organisms. Conversely, insufficient moisture content leads to the death of the mushroom. The optimal humidity for growth varies depending on the species [35,80].

### 2.3. Substrate Treatment

In mushroom cultivation, different species exhibit distinct preferences for their growth media. Mushrooms can thrive on either composted or non-composted substrates, with the suitability depending on the species. Notably, *Agaricus bisporus* serves as a prominent example of a mushroom that exclusively grows on composted substrate [34]. On the other hand, *Pleurotus* sp. can be cultivated on a non-composted medium under sterile conditions.

To achieve sterility, thermal treatment of the substrate is undertaken to eliminate potential competitors, such as bacteria, fungi, or other organisms. Depending on the mushroom species, either pasteurization or sterilization can be employed, with autoclave sterilization being the most prevalent method for substrate treatment [81]. 

According to Oseni et al. [82], the existing references on the temperature and time for heat treatments vary widely, and inadequate thermal treatment of substrate promotes the growth of fungi that compete with *Pleurotus* sp., such as *Penicillium* sp. and *Trichoderma* sp.

Beyond influencing the risk of infections, substrate treatment also affects mycelial development and mushroom quality. Yang et al. [51] reported a higher mycelial growth rate, a shorter incubation period, and quicker basidioma formation for a non-sterilized substrate. However, this did not translate to a superior mushroom yield or biological efficiency. Instead, mushrooms from the non-sterilized substrate exhibited lower quality, characterized by a smaller cap diameter and longer stipe length [51].

### 2.4. Inoculation and Incubation

After cooling the sterilized substrate, the spawn is incorporated (inoculation). The amount used for inoculation affects the duration of colonization/incubation and, consequently, fruiting [83]. The added amount of spawn should not exceed 10% of the weight of the substrate [84] and should be up to 5% of the wet weight [85]. Subsequently, the substrate bags with the spawn are stored in a dark place under the appropriate environmental conditions (Table 3).

With the beginning of substrate colonization, there is an increase in the enzymatic activity of laccase and Mn-peroxidase [86]. During this phase, it is important to ensure optimal conditions for the mycelium to be able to colonize the substrate in the shortest possible time to reduce the window of opportunity for competition with other organisms [35].

As a rule, for mushrooms, if there is more nitrogen than carbon in the substrate, the inhibition of mycelial growth occurs, despite the increase in laccase activity. However, if there is a very high C:N ratio, the incubation rate also decreases because the nitrogen deficit also has an inhibitory effect on mycelial growth [35,51]. When the substrate is completely colonized, in the case of *Pleurotus ostreatus*, a cold shock of 5–10 °C is applied to promote fructification [84,87]. The incubation stage ends when mushroom primordia appear in the bags of substrate. These bags are transported to a fruiting chamber, where the environmental conditions will be different. 

Table 3 presents the production parameters and culture duration for *P. ostreatus* according to Stamets [83]. However, it is noteworthy that these parameters may vary. According to Hu et al. [88], mycelial growth differed significantly at different test temperatures. A temperature of 22 °C was found to be optimal for the mycelial development of *P. ostreatus*. However, Hoa et al. [77] investigated the effects of various temperatures on mycelial growth and observed that the optimal temperature for such growth was 28 °C.

Furthermore, according to Oei et al. [84], some varieties of *P. ostreatus* can be cultivated at a temperature near 30 °C. The *Pleurotus ostreatus* variety *Florida* can tolerate elevated temperatures, in contrast to the standard *P. ostreatus*. Characterized by a flat, white pileus, this variety thrives during the spawn run phase at temperatures between 22 and 28 °C, with an optimal temperature of 24 °C. Basidioma development is typically observed within the temperature range of 15–22 °C [89].

Additionally, the total cropping time may be affected by the composition of the substrate, as demonstrated by Girmay et al. [53]. Their study concluded that the maturity of *P. ostreatus* could be achieved in 27 days when cultivated in cottonseed, compared to approximately 40 days with wheat straw. Similarly, Hoa et al. [77] reported that the total colonization period lasted 30 days in sugarcane bagasse and 40 days in corncobs.

### 2.5. Fructification

The onset of the fruiting phase is marked by the appearance of the mushroom primordia, which will grow and develop to form the basidioma. To ensure optimal development, the precise control and maintenance of specific temperature, relative humidity, and CO_2_ concentration values are essential, as referenced in Table 3. Certain cultivated species, such as the globally produced white button mushroom (*A. bisporus*), necessitate the presence of a casing layer on the colonized substrate to stimulate the formation of the mushroom basidioma [90].

In the case of *Pleurotus*, primordia are not highly responsive to these stimuli if the substrate is not fully colonized. In such instances, the mycelium persists in attempting to colonize the entire substrate, causing a delay in the fruiting process [83].

Once in the fruiting stage, a reduction in CO_2_ concentration is required, as well as an increase in O_2_, since high CO_2_ levels will produce mushrooms with both thick and short stipes and caps [80].

In addition to the parameters mentioned above, luminosity is a crucial factor to control, as it plays a role in inducing fruiting. According to Nakano et al. [91], certain mushrooms, including *Pleurotus* spp., require light for primordia formation, and both the wavelength and luminosity intensity exert an influence on growth and morphogenesis. 

As mentioned by Oei et al. [84], mushrooms’ shapes give information about whether they have received sufficient light and aeration. Inadequate light, as well as inappropriate aeration, leads to abnormal mushroom growth with undesirable morphological impacts, such as cap atrophy and stem elongation. Oyster mushrooms cultivated in darkness will form no caps, only stipes with a coral-like morphology. Zawadzka et al. [92] concluded that, besides its impact on morphology, light intensity has a major impact on bioactive component contents, such as phenolics, thiamine, and riboflavin. Moreover, light intensity is a critical factor influencing the vitamin D content of mushrooms. Despite their high levels of ergosterol, the precursor to vitamin D_2_, mushrooms typically contain low levels of vitamin D. As demonstrated by Gallotti and Lavelli [93], UV irradiation (0.4 W/m^2^) induces the conversion of ergosterol into vitamin D_2_ in both fresh and dried mushrooms. This process leads to a substantial increase in the vitamin D_2_ content of *P. ostreatus* mushrooms, elevating it from 3.1 to 37 µg/g of dry weight.

Another important factor in fruiting is the pH, with the optimal range falling between 3.5 and 5.0 [80]. This is a lower value than the substrate’s initial pH, but during incubation, there is degradation of substrate compounds and the production of organic acids that acidify the substrate, naturally lowering the pH [77].

In addition to the need for air humidity regulation, it is also necessary to control the water content of the substrate, since a deficit of water causes the dehydration of the mycelium, with consequences for the mushroom [35].

At this stage, there may be several fruiting periods interspersed with production breaks that last a few days, in which the mycelium metabolizes compounds again, forming new beginnings that will bear fruit. At this point, the C:N ratio continues to have an impact on fruiting. Furthermore, the yield of second flushes is closely tied to the availability of remaining simple carbon from the first flush [10].

The study conducted by Jin et al. [67] reported that corncobs supplemented with herbs (C:N = 36.9), in comparison with a control substrate consisting solely of corncobs (C:N = 53.4), exhibited a higher yield, approximately 978 g compared to 856 g, within a 2.5 kg substrate formulation. In Hoa et al. [77], each bag of 1 kg substrate produced 256 g on sugarcane bagasse and 271 g on corncobs. 

The utilization of different substrates exerts a considerable influence on the mushroom yield. Further research aimed at refining cultivation formulas is essential for optimizing the yield of *P. ostreatus*.

## 3. Mushroom Postharvest Activities

Typically, mushrooms have a high moisture content, ranging from 85 to 95% of their fresh weight. The water content in mushrooms can vary significantly, influenced by factors such as the time of harvest, misting during cultivation, postharvest conditions, and the temperature and relative humidity experienced during growth [94]. When the mushrooms are ready to be harvested, the shape of the cap changes from a convex shape to a more or less flat edge at the margins.

After harvesting, mushrooms are quite perishable, with a tendency to lose moisture and firmness and become susceptible to enzymatic browning. Given their high metabolic rate and the absence of a protective cuticle, the product undergoes rapid weight loss, limiting its storage time [95,96], which introduces a new set of concerns.

In response to these challenges, it becomes imperative to implement postharvest processing mechanisms aimed at enhancing product durability and mitigating potential losses in both quality and economic value. The inherent challenges in storing and transporting mushrooms, particularly *Pleurotus ostreatus*, come to the forefront.

**Figure 5:**
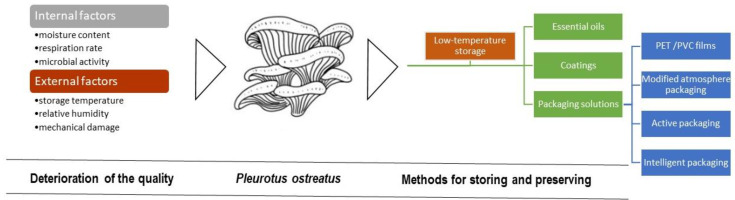
Postharvest quality degradation and methods of storing and preserving *P. ostreatus*.

Mushrooms are not suitable for long-term storage or long-distance transportation. Therefore, they must be sold fresh in a short period and cannot be kept at room temperature for more than 24 h [97]. However, the increased consumption of these products requires improved and preferably environmentally friendly preservation methods. Therefore, postharvest practices play a crucial role in maintaining the quality, safety, and marketability of mushrooms. The upcoming section emphasizes the specific deterioration factors affecting the quality of *Pleurotus ostreatus* and the importance of developing improved and environmentally friendly preservation methods to maintain quality and extend shelf-life, also addressing the challenges associated with the storage and packaging of fresh mushrooms (Figure 5).

### 3.1. Deterioration of the Quality of Pleurotus ostreatus

Fresh mushrooms are highly perishable. The loss of quality is characterized by a reduction in sensory and nutritional quality caused by internal factors (moisture content, respiration rate, and microbial activity) and external factors (storage temperature, relative humidity, and mechanical damage) [98].

The most important characteristic of the mushroom’s metabolism is its high respiratory rate (200–500 mg/kg h at 20 °C) and high moisture content, so, along with the absence of a protective barrier against water loss, they tend to lose moisture rapidly and to be susceptible to bacterial deterioration [99,100]; therefore, the mushroom respiration rate is an index of their shelf-life [101]. The absence of a protective layer, high polyphenol oxidase activity, phenolic compound contents, pathogens, and external factors such as temperature and relative humidity make them susceptible to browning [102,103], with color being one of the most important commercial quality attributes influencing the consumer’s purchase decision [97,103].

As mentioned above, relative humidity is another factor that influences the quality of the mushroom after harvest. When mushrooms are exposed to very high relative humidity, it promotes water condensation on the mushroom surface, which accelerates microbial growth and discoloration [104]. If they are exposed to low levels of surrounding humidity, it causes an excessive loss of weight, where a loss of 5–10% of their fresh weight makes them unsuitable for commercial sale [100], and a loss of firmness. After harvest, the common firm texture of mushrooms becomes spongy and tough [105].

The growth of pathogens, as already mentioned, is also one of the factors that influence the quality of the product. After storage, the bacterial load of mushrooms tends to increase, where *Pseudomonas* spp. are the most prevalent group [106]. This genus comprises *Pseudomonas tolaasii*, the most frequent agent causing brown blotch disease and the yellowing of *Pleurotus ostreatus* [107,108].

With a comprehensive understanding of the factors contributing to the postharvest deterioration of *Pleurotus ostreatus*, our exploration extends to the methods designed to counteract these challenges. 

### 3.2. Methods for Storing and Preserving Pleurotus ostreatus

Nowadays, some preservation techniques are associated with food nutritional content degradation. The effects on consumer health have led to a demand for alternative and natural food preservatives [109].

Various techniques are employed for preserving mushrooms and extending their shelf-life. The application of essential oils and coatings represents alternative preservation techniques that are environmentally friendly and prioritize consumer health. However, the most common preservation methods involve various packaging solutions [110], which can vary greatly in terms of effectiveness and cost. Regardless of the method used, the application of low-temperature storage is essential to minimize losses [111]. The following sections will explore these methods for maintaining postharvest quality during storage, including low-temperature storage [112], essential oils (EOs), and edible coatings formulated with natural substances [113].

#### 3.2.1. Low-Temperature Storage

The temperature and storage time are crucial to maintaining the quality of the mushrooms once they influence the respiration rate [112]. Typically, the higher the temperature, the higher the respiration rate of postharvest mushrooms, which leads to quality deterioration. Dama et al. [114] observed that as temperature increases, antioxidative enzyme activities, such as superoxide dismutase and peroxidase, also increase, as they are involved in countering oxidative stress conditions, which may be the main cause of fungal decay.

For most mushrooms, at a temperature of 0 °C, the storage time can be up to 21 days; at 2 °C, 8–11 days; at 5 °C, 4–6 days; at 10 °C, 2–3 days; and at 20 °C, about 1–2 days [115]. Regarding *P. ostreatus*, it can be stored for 1–2 days at room temperature (20 °C), 5–7 days in refrigeration (4 °C), and 8–11 days at 0 °C [27].

According to Azevedo et al. [112], relative humidity is also a factor to consider. To reduce weight loss, low temperatures and high relative humidity are ideal. The lower the temperatures during postharvest, the less the weight loss. On the other hand, at a higher storage temperature, there is a greater loss of water from the mushrooms through transpiration.

Although refrigeration is one of the most widely used preservation techniques to increase the shelf-life of products, it has limitations in controlling mushroom browning [105], and some mushroom species are sensitive to low temperatures [116]; therefore, other preservation techniques are needed.

#### 3.2.2. Essential Oil Treatment

Essential oils (EOs) are volatile compounds extracted from different parts of plants, such as leaves, flowers, and other plant tissues [117]. These substances are classified by the United States Food and Drug Administration (FDA) as generally recognized as safe (GRAS) and have a low resistance induction effect in pathogenic microorganisms [118]. While EOs are primarily used in the food industry as flavorings, they represent a noteworthy source of natural antimicrobials for food preservation.

The utilization of EOs is gaining prominence in enhancing the quality of freshly harvested mushrooms, being an alternative to conventional preservation methods. They have been used in food preservation to extend shelf-life because of their variety of constituents (e.g., terpenes, terpenoids, carotenoids, coumarins) with antibacterial and antifungal properties [119]. Some studies have revealed the positive effects of essential oil usage on the preservation of different food matrices, such as meat [120], fish [121], fruits, and vegetables [122,123]. Nevertheless, the efficacy of an EO as a preservative relies on its interactions with components in the food matrix, such as fat or protein, and is influenced by factors like pH, temperature, and contamination levels [124].

Consequently, various studies have investigated the application of essential oils (EOs) for preserving mushrooms. In general, the use of EOs prevents mushroom browning [108,110], maintains sensory quality, decreases bacterial counts, increases antioxidant activity, and maintains the total phenolic compounds and flavonoid content during storage [94,101,109]. The adoption of EO treatments presents a promising approach to increasing the durability and excellence of mushrooms and diminishing economic losses. Their mode of action in preserving mushrooms seems to involve several mechanisms (e.g., antimicrobial properties, disruption of cell membranes, pH modulation, antioxidant activity) that vary depending on the EO or its constituents, and each component in the oil can provide insights into its properties [125]. For example, lavender and peppermint EOs seem to function as effective tyrosinase inhibitors, disrupting enzymatic activities and thereby preventing mushroom browning [126].

Beyond their preservation benefits, EOs have also been investigated for their potential to enhance the nutritional quality (vitamins C and D_2_) of stored mushrooms [127]. This represents a potential avenue for developing functional and nutritionally enriched food.

Nevertheless, despite the demonstrated potential of EOs, their application as practical food preservatives is constrained by the need for elevated concentrations to attain substantial antimicrobial efficacy. This limitation may result in an organoleptic impact, as the use of natural preservatives has the potential to alter the taste of food and surpass the flavor threshold deemed acceptable to consumers [128].

EOs can be introduced into the product through fumigation; however, several methods can be employed to mitigate flavor concerns. They can be seamlessly integrated into polymers for edible coatings, allowing for their gradual release onto the food surface. Alternatively, EOs can be utilized in MAP rather than being directly applied to the product [129], as illustrated in Table 4. A compelling strategy to reduce the required doses of EOs involves exploring the utilization of edible coatings as carriers for these natural compounds.

It is important to note that the efficacy of essential oils as preservatives may vary depending on factors such as the composition of the essential oil, the concentration used, and the specific microorganisms targeted. Additionally, research in this field continues to explore optimal formulations and application methods to maximize the preservation benefits while ensuring product safety and consumer acceptance [131].

#### 3.2.3. Coatings

Edible coatings are biopolymer-based packaging materials that may be consumed after food application [113]. They are applied as a thin layer on the product’s surface, acting as semipermeable barriers to oxygen (O_2_), carbon dioxide (CO_2_), and humidity, leading to a modified internal atmosphere. Consequently, they contribute to reduced respiratory rates, delayed ripening, minimized weight loss, and the preservation of mushroom firmness, freshness, color, and bioactive compounds [97,132,133]. Additionally, edible coatings play a crucial role in retaining flavor compounds and nutritional content, thereby enhancing antioxidant capacity [95].

As concerns about the safety of preservatives have risen, there has been a growing interest in natural coatings with antimicrobial properties. Recent years have witnessed extensive research on various types of edible coatings to prolong the storage life of mushrooms. Key substances used for these coatings include chitosan, alginate, pectin, carrageenan, cellulose and starch derivatives, agar, and gums [133].

Chitosan and alginate emerge as promising substances for coatings in food preservation. Many studies have evaluated their individual impact on postharvest quality or assessed their combined effectiveness with other compounds [134]. The incorporation of these polysaccharides with essential oils (EOs) to formulate edible coatings has gained attention due to their gradual release on the product surface over time [118].

Research indicates that edible chitosan-based coatings enriched with rosemary and lavender essential oils exhibit antimicrobial and antioxidant properties [135]. Furthermore, they reduce the water vapor permeability and lipid oxidation of the product [136]. According to several authors, both chitosan and alginate, when enhanced with essential oils, contribute to maintaining firmness, stabilizing respiration rates, reducing microorganisms, and improving the phytochemical content of various mushroom species [137,138]. Shenbagam et al. [139] explored the use of Aloe Vera gel-based edible coatings with orange peel essential oil, reporting an improvement in postharvest quality and an extension of shelf-life.

Moreover, the application of coatings has proven advantageous, enabling the integration of diverse components, such as essential oils. This integration not only extends the shelf-life of mushrooms but also mitigates the risk of pathogen development on their surfaces [119]. The synergies between coatings and EOs as preservation methods become increasingly apparent, offering a comprehensive approach to enhance the overall quality and longevity of *Pleurotus ostreatus*.

The choice of coating depends on factors such as the specific requirements of the mushrooms, the desired shelf-life extension, and consumer preferences. The development of effective coatings involves considering the permeability, adhesion, and sensory attributes to ensure that the coated mushrooms meet both quality and safety standards.

#### 3.2.4. Packaging Solutions

Suitable packaging stands as a crucial method for maintaining the quality and prolonging the shelf-life of mushrooms. Mushrooms are typically packaged in plastic films, such as polyethylene terephthalate (PET) or polyvinyl chloride (PVC), or wrapped with PVC film or other stretchable films. However, alternative materials have emerged, including PET with varying degrees of perforation and materials derived from renewable resources, such as poly(lactic acid)/poly(ε-caprolactone) blend films and wheat gluten (WG)-coated paper [140,141]. Research indicates that WG-coated paper is particularly effective in improving the shelf-life of mushrooms compared to commonly used stretchable PVC film used for over-wrapping [141].

Modified atmosphere packaging (MAP) represents a straightforward and cost-effective method for regulating physiological effects and microbial growth in mushrooms [140,141]. MAP entails altering the atmosphere within the package, influenced by the product’s respiration rate and gas transfer through the packaging material [142]. The efficacy of MAP’s storage effect can be influenced by various factors, including packaging materials, gas composition, storage temperature and humidity, and the surface area of the packaged sample [141]. A reduced concentration of O_2_ holds the potential for diminishing mushroom respiration rates and controlling physiological effects like color and texture changes, as well as microbial growth [140]. Some studies advocate for an atmosphere with low O_2_ content (ranging from 2% to 10%) and limited CO_2_ content (not exceeding 5%). Conversely, experiments with high O_2_ concentrations (up to 80%) for button mushrooms have shown benefits such as lower lipid peroxidation rates and the reduced production of reactive oxygen species [140,141,143]. The selection of suitable packaging material is crucial for maintaining the quality of packaged products. Different materials may be chosen based on storage conditions (refrigerated or room temperature), mushroom presentation type (sliced or whole), and packaging technology (with or without MAP, type of MAP) [140]. For instance, MAP combined with low-temperature storage proves effective in enhancing the shelf-life of fresh mushrooms. Moreover, microperforated packaging films are commonly employed to mitigate CO_2_ accumulation, O_2_ depletion, water condensation, and elevated humidity levels, all of which hasten microbial growth and browning [143].

In addition to the methodologies mentioned above, the recent advancements in active packaging offer another avenue to prolong the freshness of mushrooms. Active packaging refers to a type of packaging that goes beyond merely containing a product; it actively interacts with the packaged product or its environment to extend shelf-life, improve safety, or enhance sensory characteristics. Unlike traditional passive packaging, which mainly serves as a barrier to external factors, active packaging incorporates active agents or components that can release, absorb, or interact with substances within the packaging environment [144]. 

Utilizing active packaging for the preservation of mushrooms holds significant relevance due to their susceptibility to spoilage caused by the different factors already mentioned. Studies have identified different active packaging strategies that effectively tackle these challenges in different mushroom varieties: incorporating components that thermally buffered the package reduced temperature fluctuations during product transport and temporary storage [145]; using plasma modification and natural polymer materials led to the development of a preservation packaging material that extended shelf-life to more 13 days [146]; and zeolites combined with an aҫai extract active coating in an active packaging system decreased water loss and browning, extending shelf life to 28 days [147]. Wrona et al. [148] incorporated four different active agents, namely, sodium metabisulphite combined with citric acid, green tea extract, cinnamon essential oil, and purple carrot extract, into packaging material to extend the shelf-life of packed mushrooms. There are few publications about the effects of active packaging on *Pleurotus ostreatus* mushroom’s storage period. Han Lyn et al. [149] investigated the effect of combining modified atmosphere packaging with bilayer active packaging (MAP  +  BL). The BL active packaging consisted of gelatin with pomegranate peel powder (PPP) coated on a polyethylene (PE) film (gelatin  +  PPP/PE). The authors also evaluated three different conditions of MAP: high-oxygen packaging (HOP), medium-oxygen packaging (MOP), and low-oxygen packaging (LOP). The authors concluded that packaging mushrooms in MOP with an active layer effectively extended the shelf-life of the mushrooms to 11 days, in contrast to the control group, which lasted only 3 days. 

Leveraging active packaging technologies, the shelf-life of mushrooms can be significantly prolonged, ensuring freshness, quality, and safety for consumers. Furthermore, active packaging can enhance the marketability of mushrooms by preserving their visual appeal and nutritional value over an extended period, thereby meeting the escalating demand for fresh and high-quality produce. Nevertheless, it is important to note that creating a new active packaging solution to extend the freshness of mushrooms, especially when they are sliced, is a challenging endeavor. Numerous obstacles and limitations must be considered, including the challenge of identifying a suitable antioxidant that is effective across various parameters, safe for consumption, and seamlessly incorporable into the packaging material.

Intelligent packaging, emerging as a cutting-edge method [150], provides real-time product tracking [151], facilitates convenient information exchange, enables the swift detection of food freshness, and boasts wide applicability [152]. This technology not only enhances food safety but also contributes to its overall healthiness. Through advanced techniques like pattern recognition and deep learning, the precise detection of food freshness can be achieved [153]. Intelligent packaging was prepared and evaluated to determine the quality of mushrooms using colorimetric sensing by Fan et al. [154] and Liu et al. [155]. However, no studies were found on intelligent packaging applied to the preservation of *P. ostreatus* mushrooms. Therefore, further research in this area is warranted to explore its potential benefits and implications. Such research has the potential to significantly enhance our understanding of how these innovative packaging technologies can extend the shelf-life, uphold the quality, and ensure the safety of mushrooms. Delving into the application of intelligent packaging for *P. ostreatus* mushrooms specifically could lead to tailored preservation solutions that cater to the unique characteristics and requirements of this variety. Moreover, by investigating the potential benefits and challenges associated with implementing these technologies, researchers can pave the way for advancements in mushroom preservation methods, thereby contributing to both industry practices and consumer satisfaction.

## 4. Circular Economy Approach: Waste Reuse

The byproducts arising from mushroom production mainly comprise two categories: spent mushroom substrate (SMS) and low-quality mushrooms or residues generated during harvesting.

Regarding the first type, after the harvesting period, for each kilogram of mushrooms produced, approximately 5 kg of SMS is generated as a byproduct [156,157]. Improper disposal methods, such as burning or landfilling, can result in water contamination, leading to eutrophication and air pollution. The composition of SMS plays a significant role in its use across different applications. It contains significant amounts of mycelia, enzymes, organic compounds such as proteins and carbohydrates, and inorganic compounds like ammonium nitrate [6].

Numerous studies have highlighted the potential of *Pleurotus* sp. SMS for various applications, including metal adsorption and pollutant and endocrine disruptor removal. Chang et al. [158] demonstrated that *Pleurotus* spp. SMS offers a practical solution for removing emerging pollutants from wastewater. Additionally, SMS from other species, such as *Agaricus* sp., has been investigated for heavy metal bioremediation in wastewater [159]. Moreover, SMS can serve as an organic soil amendment to improve soil fertility and quality [160]. Organic amendments like straw, compost, biochar, and manure have been successfully utilized for treating cadmium-contaminated soils [161]. However, SMS presents itself as a more cost-effective and environmentally friendly option. According to García-Delgado et al. [162], SMS demonstrates exceptional potential in mitigating heavy metal contamination and biodegrading polycyclic aromatic hydrocarbons. Their findings suggest that SMS effectively immobilizes heavy metals, such as cadmium and lead, thereby reducing their bioavailability in the soil. Furthermore, SMS microbial activity enhances the degradation of pollutants.

Additionally, SMS can be efficiently recycled in mushroom cultivation. Research suggests that substrates from *Pleurotus* spp. production can be repurposed for cultivating mushrooms of the same genus [163] and, when transformed into biochar, can enhance yields and shorten cultivation periods compared to conventional methods [164].

Fungal enzymes, including laccases and peroxidases, are crucial in biotechnological applications. Efforts have been made to utilize SMS as a cost-effective source of ligninolytic enzymes for industrial purposes [165,166,167]. For instance, Branà et al. [168] found that ligninolytic enzymes derived from *Pleurotus* spp. SMS were effective in degrading up to 90% of aflatoxin B1, a known carcinogen, in food commodities. Additionally, it has been estimated that up to 30% of SMS disposal could be reduced by using it for cellulolytic enzyme production [166].

Another way of using SMS is in animal feeding. Baptista et al. [169] identified the use of SMS for feeding insects, rabbits, pigs, and ruminants. It can be fermented using specific microbial inoculants and transformed into silages with increased nutrient digestibility and quality for animal use [170,171].

Numerous studies have demonstrated the feasibility of utilizing SMS in horticultural applications. Its application alters the soil structure and porosity [172], enhances the mineral nitrogen content in soil [173], and acts as a biological control [174]. Furthermore, Huang et al. [175] transformed SMS into a liquid fertilizer with great results in the production of Pak-choi cabbage, with about 30% more productivity and improved nutrients in the soil. It can also be combined with manure and be used as a growing medium for seedlings [176]. Beyond the almost endless possibilities of applications in agriculture, *Pleurotus* spp. SMS holds substantial promise in the realm of renewable energy production as a source of biogas [177], bioethanol [178], and solid biofuels [179], such as briquettes. 

In relation to mushroom byproducts, suboptimal environmental conditions and substrate composition during the production process may yield mushrooms of inferior quality. Furthermore, in the process of mushroom harvesting, the lower portion of the stem often remains within the substrate [180]. This residual section has found application as an ingredient in several products, such as chicken patties [180], cookies, steamed buns [181], and noodles [182]. Notably, these products collectively exhibit an antioxidant effect, thereby highlighting the potential value of repurposing mushroom byproducts as functional ingredients in the formulation of functional foods.

Furthermore, mushroom byproducts can serve as ingredients to enhance the flavor of various food products, enabling salt reduction [183] and improving shelf-life stability. They also exhibit properties in meat products, such as inhibiting lipid oxidation and retarding the growth of spoilage bacteria during storage [184,185,186]. Several other studies explored the use of mushrooms as meat analogs and replacers [187,188], replacing fat and enhancing the nutritional profile. Additionally, mushrooms can also be incorporated as flour into breads, replacing wheat flour, thereby enhancing fiber content and improving protein content and bioactive compounds [18].

Despite the promising outcomes of utilizing mushrooms as functional ingredients and additives, few studies have specifically addressed the utilization of mushroom byproducts, particularly those from the *Pleurotus ostreatus* species.

## 5. Conclusions

The mushroom industry, particularly the cultivation of *Pleurotus ostreatus*, emerges as a promising avenue for transforming byproducts into valuable resources. With its adaptability and ability to utilize various substrates, *P. ostreatus* stands out as a key player in sustainable agriculture. Successful mushroom cultivation demands a comprehensive understanding of the intricate interactions between environmental factors, substrate composition, and the life cycle of the mushrooms. This knowledge is crucial for maximizing productivity, ensuring product quality, and promoting sustainability in mushroom cultivation practices. However, this review points out a gap in the literature concerning postharvest strategies and health-conscious preservation methods for *P. ostreatus*. Future research should focus on implementing environmentally conscious postharvest strategies. In summary, successful *Pleurotus ostreatus* cultivation requires a holistic approach, integrating precise cultivation practices with effective pre- and postharvest techniques. The ongoing exploration of environmentally friendly methods aligns with the demand for fresh, high-quality mushrooms in the market.

## Figures and Tables

**Table 1:** Pleurotus ostreatus taxonomic description.

Taxonomic Rank	Classification
Kingdom	Fungi
Phylum	Basidiomycota
Class	Agaricomycetes
Order	Agaricales
Family	*Pleurotaceae*
Genus	*Pleurotus*
Species	*Pleurotus ostreatus*

**Table 3:** Production parameters for *P. ostreatus* and culture duration according to Stamets [83].

Parameters	Incubation	Primordia Formation	Basidioma Development
Temperature (°C)	24	10–15	10–21
RH (%)	85–95	95–100	85–90
[CO_2_] (ppm)	5000–20,000	<1000	<1000
Time (days)	12–21	3–5	4–7
Luminosity (Lux)	-	1000–1500	1000–1500

**Table 4:** EOs used in the preservation of mushrooms.

Essential Oil	Mushroom Species	Research Outcome	References
Clove and peppermint EOs	*Volvariella volvacea*	The active antioxidant packaging composed of EOs was effective mainly with peppermint oil.	[116]
Eugenol, bergamot, and grapefruit EOs	*Agaricus bisporus*	Vaporized EOs within the MAP reduce the quality loss of sliced mushrooms during postharvest storage.	[105]
Clove, cinnamaldehyde, and thyme EOs	*Lentinula edodes*	EO fumigation maintained sensory quality during storage and increased AOx.	[130]
Cinnamon EO	*Agaricus bisporus*	Bioactive food packaging was suitable for extending the shelf-life of high-moisture-content products.	[16]

## Data Availability

No new data were created or analyzed in this study. Data sharing is not applicable to this article.

## References

[1] Gaur V.K., Sharma P., Sirohi R., Awasthi M.K., Dussap C.G., Pandey A. (2020). Assessing the impact of industrial waste on environment and mitigation strategies: A comprehensive review. J. Hazard. Mater..

[2] Correddu F., Lunesu M.F., Buffa G., Atzori A.S., Nudda A., Battacone G., Pulina G. (2020). Can Agro-Industrial By-Products Rich in Polyphenols be Advantageously Used in the Feeding and Nutrition of Dairy Small Ruminants?. Animals.

[3] Sharmin Z., Noor R.M., Soon T.K., Ahmedy I., Abdullah N.A., Poh Y.S. IoT Based Multidimensional Mushroom Waste Management System in Urban Area. Proceedings of the 3rd International Conference on Sustainable Technologies for Industry 4.0 STI 2021.

[4] Comunicação da Comissão ao Parlamento Europeu, ao Conselho, ao Comité Económico e Social Europeu e ao Comité das Regiões Sobre o Regime de Acompanhamento do 8.° Programa de Ação em Matéria de Ambiente: Medir os Progressos Realizados para Alcançar os Objetivos Prioritários do Programa para 2030 e 2050. https://eur-lex.europa.eu/legal-content/PT/TXT/PDF/?uri=CELEX:52022DC0357&from=EN.

[5] Royse D.J., Baars J., Tan Q., Diego C.Z., Pardo-Giménez A. (2017). Current Overview of Mushroom Production in the World. Edible and Medicinal Mushrooms: Technology and Applications.

[6] Wan Mahari W.A., Peng W., Nam W.L., Yang H., Lee X.Y., Lee Y.K., Liew R.K., Ma N.L., Mohammad A., Sonne C. (2020). A review on valorization of oyster mushroom and waste generated in the mushroom cultivation industry. J. Hazard. Mater..

[7] Singh M.P., Singh V.K. Yield performance and nutritional analysis of *Pleurotus citrinopileatus* on different agrowastes and vegetable wastes. Proceedings of the 7th International Conference on Mushroom Biology and Mushroom Products (ICMBMP7).

[8] Barshteyn V., Krupodorova T. (2016). Utilization of agro-industrial waste by higher mushrooms: Modern view and trends. J. Microbiol. Biotechnol. Food Sci..

[9] Abou Fayssal S., El Sebaaly Z., Alsanad M.A., Najjar R., Böhme M., Yordanova M.H., Sassine Y.N. (2021). Combined effect of olive pruning residues and spent coffee grounds on *Pleurotus ostreatus* production, composition, and nutritional value. PLoS ONE.

[10] Akter M., Halawani R.F., Aloufi F.A., Taleb M.A., Akter S., Mahmood S. (2022). Utilization of Agro-Industrial Wastes for the Production of Quality Oyster Mushrooms. Sustainability.

[11] Zhou F., Hansen M., Hobley T.J., Jensen P.R. (2022). Valorization of Green Biomass: Alfalfa Pulp as a Substrate for Oyster Mushroom Cultivation. Foods.

[12] Bach F., Helm C.V., Bellettini M.B., Maciel G.M., Haminiuk C.W.I. (2017). Edible mushrooms: A potential source of essential amino acids, glucans and minerals. Int. J. Food Sci. Technol..

[13] Yadav D., Negi P.S. (2021). Bioactive components of mushrooms: Processing effects and health benefits. Food Res. Int..

[14] Jayachandran M., Xiao J., Xu B. (2017). A Critical Review on Health Promoting Benefits of Edible Mushrooms through Gut Microbiota. Int. J. Mol. Sci..

[15] Kibar B. (2021). Influence of different drying methods and cold storage treatments on the postharvest quality and nutritional properties of *P. ostreatus* mushroom. Turk. J. Agric. For..

[16] Shao P., Yu J., Chen H., Gao H. (2021). Development of microcapsule bioactive paper loaded with cinnamon essential oil to improve the quality of edible fungi. Food Packag. Shelf Life.

[17] Lou Z., Sun Y., Bian S., Ali Baig S., Hu B., Xu X. (2017). Nutrient conservation during spent mushroom compost application using spent mushroom substrate derived biochar. Chemosphere.

[18] Lu X., Brennan M.A., Serventi L., Brennan C.S. (2018). Incorporation of mushroom powder into bread dough—Effects on dough rheology and bread properties. Cereal Chem..

[19] Lu X., Brennan M.A., Narciso J., Guan W., Zhang J., Yuan L., Serventi L., Brennan C. (2020). Correlations between the phenolic and fibre composition of mushrooms and the glycaemic and textural characteristics of mushroom enriched extruded products. LWT.

[20] Antunes F., Marçal S., Taofiq O., Morais A.M.M.B., Freitas A.C., Ferreira I.C.F.R., Pintado M. (2020). Valorization of Mushroom By-Products as a Source of Value-Added Compounds and Potential Applications. Molecules.

[21] Haneef M., Ceseracciu L., Canale C., Bayer I.S., Heredia-Guerrero J.A., Athanassiou A. (2017). Advanced Materials From Fungal Mycelium: Fabrication and Tuning of Physical Properties. Sci. Rep..

[22] Wang W., Zhang K., Li C., Cheng S., Zhou J., Wu Z. (2018). A Novel Biodegradable Film from Edible Mushroom (*F. velutipes*) By-Product: Microstructure, Mechanical, and Barrier Properties Associated with the Fiber Morphology. Innov. Food Sci. Emerg. Technol..

[23] Sahithya K., Mouli T., Biswas A., Mercy S. (2022). Remediation Potential of Mushrooms and Their Spent Substrate Against Environmental Contaminants: An Overview. Biocatal. Agric. Biotechnol..

[24] van Eck N.J., Waltman L. (2010). Software survey: VOSviewer, a computer program for bibliometric mapping. Scientometrics.

[25] Zhang J., Yu Q., Zheng F., Long C., Lu Z., Duan Z. (2016). Comparing Keywords Plus of WOS and Author Keywords: A Case Study of Patient Adherence Research. J. Assoc. Inf. Sci. Technol..

[26] Kotadiya U., Talaviya J., Shah K., Lathiya S. (2021). Morphological and Molecular Identification of Oyster Mushroom [*Pleurotus ostreatus* (Jacq.) P. Kumm]. Res. Sq..

[27] Raman J., Jang K.Y., Oh Y.L., Oh M., Im J.H., Lakshmanan H., Sabaratnam V. (2020). Cultivation and Nutritional Value of Prominent *Pleurotus* spp.: An Overview. Mycobiology.

[28] Kozarski M., Klaus A., van Griensven L., Jakovljevic D., Todorovic N., Wan-Mohtar W.A.A.Q.I., Vunduk J. (2023). Mushroom β-glucan and polyphenol formulations as natural immunity boosters and balancers: Nature of the application. Food Sci. Hum. Wellness.

[29] Rennerova Z., Picó Sirvent L., Carvajal Roca E., Paśnik J., Logar M., Milošević K., Majtan J., Jesenak M. (2022). Beta-(1,3/1,6)-D-glucan from *Pleurotus ostreatus* in the prevention of recurrent respiratory tract infections: An international, multicentre, open-label, prospective study. Front. Pediatr..

[30] Cateni F., Gargano M.L., Procida G., Venturella G., Cirlincione F., Ferraro V. (2022). Mycochemicals in Wild and Cultivated Mushrooms: Nutrition and Health. Phytochem. Rev..

[31] Bell V., Silva C.R.P.G., Guina J., Fernandes T.H. (2022). Mushrooms as future generation healthy foods. Front. Nutr..

[32] Di Piazza S., Benvenuti M., Damonte G., Cecchi G., Mariotti M.G., Zotti M. (2021). Fungi and circular economy: *Pleurotus ostreatus* grown on a substrate with agricultural waste of lavender, and its promising biochemical profile. Recycling.

[33] Zhang R.Y., Hu D.D., Ma X.T., Li S.G., Gu J.G., Hu Q.X. (2014). Adopting Stick Spawn Reduced the Spawn Running Time and Improved Mushroom Yield and Biological Efficiency of *Pleurotus eryngii*. Sci. Hortic..

[34] Suwannarach N., Kumla J., Zhao Y., Kakumyan P. (2022). Impact of Cultivation Substrate and Microbial Community on Improving Mushroom Productivity: A Review. Biology.

[35] Bellettini M.B., Fiorda F.A., Maieves H.A., Teixeira G.L., Ávila S., Hornung P.S., Júnior A.M., Ribani R.H. (2019). Factors affecting mushroom *Pleurotus* spp. Saudi J. Biol. Sci..

[36] Lee B.J., Lee M.A., Kim Y.G., Lee K.W., Lee B.E., Seo G.S. (2014). Characteristics and suitability of various cereal grains in spawn production of button mushroom. J. Mushrooms.

[37] Anusiya G., Gowthama Prabu U., Yamini N.V., Sivarajasekar N., Rambabu K., Bharath G., Banat F. (2021). A review of the therapeutic and biological effects of edible and wild mushrooms. Bioengineered.

[38] Aditya N., Jarial R.S., Jarial K., Bhatia J.N. (2024). Comprehensive review on oyster mushroom species (Agaricomycetes): Morphology, nutrition, cultivation and future aspects. Heliyon.

[39] Adebayo E.A., Elkanah F.A., Afolabi F.J., Ogundun O.S., Alabi T.F., Oduoye O.T. (2021). Molecular characterization of most cultivated Pleurotus species in sub-western region Nigeria with development of cost effective cultivation protocol on palm oil waste. Heliyon.

[40] Ma L., Lin Y.Q., Yang C., Ying Z.H., Jiang X.L. (2016). Production of Liquid Spawn of an Edible Mushroom, Sparassis latifolia, by Submerged Fermentation and Mycelial Growth on Pine Wood Sawdust. Sci. Hortic..

[41] Liu S.R., Zhang W.R., Kuang Y.B. (2018). Production of stalk spawn of an edible mushroom (*Pleurotus ostreatus*) in liquid culture as a suitable substitute for stick spawn in mushroom cultivation. Sci. Hortic..

[42] Zhang W.R., Liu S.R., Kuang Y.B., Zheng S.Z. (2019). Development of a Novel Spawn (Block Spawn) of an Edible Mushroom, *Pleurotus ostreatus*, in Liquid Culture and Its Cultivation Evaluation. Mycobiology.

[43] Gupta S., Kumar S., Singh R., Summuna B. (2020). Management of contaminants in mushroom spawn. Indian J. Agric. Sci..

[44] Abdullah N., Ismail R., Johari N.M.K., Annuar M.S.M. (2013). Production of liquid spawn of an edible grey oyster mushroom, *Pleurotus pulmonarius* (Fr.) Quél by submerged fermentation and sporophore yield on rubber wood sawdust. Sci. Hortic..

[45] Meyer V., Basenko E.Y., Benz J.P., Braus G.H., Caddick M.X., Csukai M., de Vries R.P., Endy D., Frisvad J.C., Gunde-Cimerman N. (2020). Growing a circular economy with fungal biotechnology: A white paper. Fungal Biol. Biotechnol..

[46] Petre M., Petre V., Petre M. (2013). Environmental Biotechnology for Bioconversion of Agricultural and Forestry Wastes into Nutritive Biomass. Environmental Biotechnology—New Approaches and Prospective Applications.

[47] Molobele I.I., Masalu R.J., Mosha P.R., Mpinda C.B. (2022). Evaluation of Enzymatic Activity during Growth of Pleurotus HK 37 on Saba comorensis Exocarp. Tanzan. J. Sci..

[48] Masevhe M.R., Soundy P., Taylor N.J. (2016). Alternative substrates for cultivating oyster mushrooms (*Pleurotus ostreatus*). S. Afr. J. Plant Soil.

[49] Singh K. (2016). Vegetable Waste—A Potent Substrate for Cultivation of *P. ostreatus*. Int. J. Res. Stud. Biosci..

[50] Philippoussis A.N., nee’ Nigam P.S., Pandey A. (2009). Production of Mushrooms Using Agro-Industrial Residues as Substrates. Biotechnology for Agro-Industrial Residues Utilisation: Utilisation of Agro-Residues.

[51] Yang W.J., Guo F.L., Wan Z.J. (2013). Yield and Size of Oyster Mushroom Grown on Rice/Wheat Straw Basal Substrate Supplemented with Cotton Seed Hull. Saudi J. Biol. Sci..

[52] Puliga F., Leonardi P., Minutella F., Zambonelli A., Francioso O. (2022). Valorization of Hazelnut Shells as Growing Substrate for Edible and Medicinal Mushrooms. Horticulturae.

[53] Girmay Z., Gorems W., Birhanu G., Zewdie S. (2016). Growth and Yield Performance of *Pleurotus ostreatus* (Jacq. Fr.) Kumm (Oyster Mushroom) on Different Substrates. AMB Express.

[54] Yang D., Liang J., Wang Y., Sun F., Tao H., Xu Q., Zhang L., Zhang Z., Ho C.T., Wan X. (2016). Tea waste: An effective and economic substrate for oyster mushroom cultivation. J. Sci. Food Agric..

[55] Nguyen T.M., Ranamukhaarachchi S.L. (2020). Effect of different culture media, grain sources and alternate substrates on the mycelial growth of *Pleurotus eryngii* and *Pleurotus ostreatus*. Pak. J. Biol. Sci..

[56] Pardo-Giménez A., Carrasco J., Roncero J.M., Álvarez-Ortí M., Zied D.C., Pardo-González J.E. (2018). Recycling of the Biomass Waste Defatted Almond Meal as a Novel Nutritional Supplementation for Cultivated Edible Mushrooms. Acta Sci. Agron..

[57] Alananbeh K., Al-Momany A. (2005). Production of Oyster Mushroom (*Pleurotus ostreatus*) on Olive Cake Agro Waste. Dirasat Agric. Sci..

[58] Alsanad M.A., Sassine Y.N., El Sebaaly Z., Abou Fayssal S. (2021). Spent coffee grounds influence on *Pleurotus ostreatus* production, composition, fatty acid profile, and lignocellulose biodegradation capacity. CYTA-J. Food.

[59] Grimm A., Eilertsen L., Chen F., Huang R., Atterhem L., Xiong S. (2021). Cultivation of *Pleurotus ostreatus* Mushroom on Substrates Made of Cellulose Fibre Rejects: Product Quality and Spent Substrate Fuel Properties. Waste Biomass Valorization.

[60] Salmones D., Mata G., Waliszewski K.N. (2005). Comparative culturing of *Pleurotus* spp. on coffee pulp and wheat straw: Biomass production and substrate biodegradation. Bioresour. Technol..

[61] Carrasco-Cabrera C.P., Bell T.L., Kertesz M.A. (2019). Caffeine metabolism during cultivation of oyster mushroom (*Pleurotus ostreatus*) with spent coffee grounds. Appl. Microbiol. Biotechnol..

[62] Gregori A., Švagelj M., Pahor B., Berovič M., Pohleven F. (2008). The Use of Spent Brewery Grains for *Pleurotus ostreatus* Cultivation and Enzyme Production. New Biotechnol..

[63] Elkanah F.A., Oke M.A., Adebayo E.A. (2022). Substrate composition effect on the nutritional quality of *Pleurotus ostreatus* (MK751847) fruiting body. Heliyon.

[64] Yolande M.E., Germaine M.J.E., Abraham N.T., Germaine Y., Marcellin M.L., Aime B.B.D., Leroy S.K.S. (2023). Impact of substrate methionine content on lovastatin potentiation and morphological parameters of *Pleurotus ostreatus*. Sci. Afr..

[65] Siwulski M., Budka A., Rzymski P., Gąsecka M., Kalač P., Budzyńska S., Magdziak Z., Niedzielski P., Mleczek P., Mleczek M. (2020). Worldwide basket survey of multielemental composition of white button mushroom *Agaricus bisporus*. Chemosphere.

[66] Golian M., Hegedűsová A., Mezeyová I., Chlebová Z., Hegedűs O., Urminská D., Vollmannová A., Chlebo P. (2022). Accumulation of Selected Metal Elements in Fruiting Bodies of Oyster Mushroom. Foods.

[67] Jin Z., Li Y., Ren J., Qin N. (2018). Yield, Nutritional Content, and Antioxidant Activity of *Pleurotus ostreatus* on Corncobs Supplemented with Herb Residues. Mycobiology.

[68] Oliveira A.P., Naozuka J. (2020). Elemental Enrichment by Cultivation: Plants and Mushrooms. Quim. Nova.

[69] Rzymski P., Mleczek M., Niedzielski P., Siwulski M., Gąsecka M. (2017). Cultivation of *Agaricus bisporus* enriched with selenium, zinc and copper. J. Sci. Food Agric..

[70] Naraian R., Sahu R.K., Kumar S., Garg S.K., Singh C.S., Kanaujia R.S. (2009). Influence of Different Nitrogen-Rich Supplements during Cultivation of *Pleurotus florida* on Corn Cob Substrate. Environmentalist.

[71] Reddy M.S., Kanwal H.K. (2022). Influence of carbon, nitrogen sources, inducers, and substrates on lignocellulolytic enzyme activities of Morchella spongiola. J. Agric. Food Res..

[72] Krupodorova T., Barshteyn V.Y., Sekan A. (2021). Review of the basic cultivation conditions influence on the growth of basidiomycetes. Curr. Res. Environ. Appl. Mycol..

[73] Laursen A. (2018). The Effect of Different Nitrogen Sources on Mycelial Growth of Oyster Mushroom, Pleurotus ostreatus—With a Review Concerning Cultivation of the Species.

[74] Akcay C., Ceylan F., Arslan R. (2023). Production of oyster mushroom (*Pleurotus ostreatus*) from some waste lignocellulosic materials and FTIR characterization of structural changes. Sci. Rep..

[75] Chang S.T., Miles P.G. (2004). Mushrooms: Cultivation, Nutritional Value, Medicinal Effect, and Environmental Impact.

[76] Cueva A.R., Bernarda M., Hernández, Niño-Ruiz A. (2017). Influence of C/N ratio on productivity and the protein contents of *Pleurotus ostreatus* grown in different residue mixtures. Rev. Fac. Cienc. Agrar..

[77] Hoa H.T., Wang C.L. (2015). The effects of temperature and nutritional conditions on mycelium growth of two oyster mushrooms (*Pleurotus ostreatus* and *Pleurotus cystidiosus*). Mycobiology.

[78] Atila F. (2019). Compositional changes in lignocellulosic content of some agro-wastes during the production cycle of shiitake mushroom. Sci. Hortic..

[79] Hossain I., Saifullah M.D., Amin R., Chakraborty R. (2018). Influence of Substrate pH and Watering Frequency on the Growth of Oyster Mushroom. Int. J. Plant Biol..

[80] Urben A.F. (2017). Produção de Cogumelos por Meio de Tecnologia Chinesa Modificada Biotecnologia e Aplicações na Agricultura e na Saúde.

[81] Kalita M.K. (2015). Impact of various sterilization methods on growth and yield of oyster mushroom (*Pleurotus florida*). Int. J. Agric. Sci..

[82] Oseni T.O., Dlamini S.O., Earnshaw D.M., Masarirambi M.T. (2012). Effect of Substrate Pre-treatment Methods on Oyster Mushroom (*Pleurotus ostreatus*) Production. Int. J. Agric. Biol..

[83] Stamets P. (2000). Growing Gourmet and Medicinal Mushrooms.

[84] Oei P., van Nieuwenhuijzen B., de Feijter J., de Zylva N. (2005). Small-Scale Mushroom Cultivation: Oyster, Shiitake and Wood Ear Mushrooms.

[85] Sánchez C. (2010). Cultivation of *Pleurotus ostreatus* and other edible mushrooms. Appl. Microbiol. Biotechnol..

[86] Savoie J.M., Salmones D., Mata G. (2007). Hydrogen Peroxide Concentration Measured in Cultivation Substrates during Growth and Fruiting of the Mushrooms *Agaricus bisporus* and *Pleurotus* spp. J. Sci. Food Agric..

[87] Shen Y., Gu M., Jin Q., Fan L., Feng W., Song T., Fangfang T., Cai W. (2014). Effects of cold stimulation on primordial initiation and yield of *Pleurotus pulmonarius*. Sci. Hortic..

[88] Hu Y., Xue F., Chen Y., Qi Y., Zhu W., Wang F., Wen Q., Shen J. (2023). Effects and Mechanism of the Mycelial Culture Temperature on the Growth and Development of *Pleurotus ostreatus* (Jacq.) P. Kumm. Horticulturae.

[89] Barh A., Sharma V.P., Thakur B., Annepu S.K., Kamal S., Bairwa R. (2020). Pleurotus species relationship and round the year cultivation in India. Mushroom Res..

[90] Carrasco J., Zied D.C., Pardo J.E., Preston G.M., Pardo-Giménez A. (2018). Supplementation in mushroom crops and its impact on yield and quality. AMB Express.

[91] Nakano Y., Fujii H., Kojima M. (2010). Identification of blue-light photoresponse genes in oyster mushroom mycelia. Biosci. Biotechnol. Biochem..

[92] Zawadzka A., Janczewska A., Kobus-Cisowska J., Dziedziński M., Siwulski M., Czarniecka-Skubina E., Stuper-Szablewska K. (2022). The effect of light conditions on the content of selected active ingredients in anatomical parts of the oyster mushroom (*Pleurotus ostreatus* L.). PLoS ONE.

[93] Gallotti F., Lavelli V. (2020). The Effect of UV Irradiation on Vitamin D2 Content and Antioxidant and Antiglycation Activities of Mushrooms. Foods.

[94] Cheung L.M., Cheung P.C.K., Ooi V.E.C. (2003). Antioxidant activity and total phenolics of edible mushroom extracts. Food Chem..

[95] Liu Q., Cui X., Song Z., Kong W., Kang Y., Kong W., Ng T.B. (2021). Coating shiitake mushrooms (*Lentinus edodes*) with a polysaccharide from Oudemansiella radicata improves product quality and flavor during postharvest storage. Food Chem..

[96] Xiao G., Zhang M., Shan L., You Y., Salokhe V.M. (2011). Extension of the shelf-life of fresh oyster mushrooms (*Pleurotus ostreatus*) by modified atmosphere packaging with chemical treatments. Afr. J. Biotechnol..

[97] Dawadi E., Magar P.B., Bhandari S., Subedi S., Shrestha S., Shrestha J. (2022). Nutritional and Post-harvest Quality Preservation of Mushrooms: A Review. Heliyon.

[98] Guo Y., Chen X., Gong P., Wang R., Qi Z., Deng Z., Han A., Long H., Wang J., Yao W. (2023). Advances in Postharvest Storage and Preservation Strategies for *Pleurotus eryngii*. Foods.

[99] Kim K.M., Ko J.A., Lee J.S., Park H.J., Hanna M.A. (2006). Effect of Modified Atmosphere Packaging on the Shelf-life of Coated, Whole, and Sliced Mushrooms. LWT-Food Sci. Technol..

[100] Castellanos-Reyes K., Villalobos-Carvajal R., Beldarrain-Iznaga T. (2021). Fresh Mushroom Preservation Techniques. Foods.

[101] Diamantopoulou P., Phillippoussis A., Hui Y.H., Özgül Evranuz E. (2015). Cultivated Mushrooms: Preservation and Processing. Handbook of Vegetable Preservation and Processing.

[102] Li T., Zhang J., Gao X., Chen J., Zheng Y., Gao Y., Qiu L. (2019). The molecular mechanism for the ethylene regulation of postharvest button mushrooms maturation and senescence. Postharvest Biol. Technol..

[103] Lin X., Sun D.W. (2019). Research Advances in Browning of Button Mushroom (*Agaricus bisporus*): Affecting Factors and Controlling Methods. Trends Food Sci. Technol..

[104] Rux G., Mahajan P.V., Geyer M., Linke M., Pant A., Saengerlaub S., Caleb O.J. (2015). Application of humidity-regulating tray for packaging of mushrooms. Postharvest Biol. Technol..

[105] López-Gómez A., Ros-Chumillas M., Navarro-Martínez A., Barón M., Navarro-Segura L., Taboada-Rodríguez A., Marín-Iniesta F., Martínez-Hernández G.B. (2021). Packaging of Fresh Sliced Mushrooms with Essential Oils Vapours: A New Technology for Maintaining Quality and Extending Shelf Life. Foods.

[106] Schill S., Stessl B., Meier N., Tichy A., Wagner M., Ludewig M. (2021). Microbiological Safety and Sensory Quality of Cultivated Mushrooms (*Pleurotus eryngii*, *Pleurotus ostreatus*, and *Lentinula edodes*) at Retail Level and Post-Retail Storage. Foods.

[107] Sajben E., Manczinger L., Nagy A., Kredics L., Vágvölgyi C. (2011). Characterization of pseudomonads isolated from decaying sporocarps of oyster mushroom. Microbiol. Res..

[108] Azu Okorley B., Leo Sossah F., Dai D., Xu S., Liu Z., Song B., Sheng H., Fu Y., Li Y. (2019). Resistance Sources to Brown Blotch Disease (Pseudomonas tolaasii) in a Diverse Collection of Pleurotus Mushroom Strains. Pathogens.

[109] Rout S., Tambe S., Deshmukh R.K., Mali S., Cruz J., Srivastav P.P., Amin P.D., Gaikwad K.K., Andrade E.H.A., de Oliveira M.S. (2022). Recent trends in the application of essential oils: The next generation of food preservation and food packaging. Trends Food Sci. Technol..

[110] Palacios I., Moro C., Lozano M., D’Arrigo M., Guillamón E., García-Lafuente A., Villares A. (2011). Use of modified atmosphere packaging to preserve mushroom quality during storage. Recent Pat. Food Nutr. Agric..

[111] Villaescusa R., Gil M. (2003). Quality improvement of Pleurotus mushrooms by modified atmosphere packaging and moisture absorbers. Postharvest Biol. Technol..

[112] Azevedo S., Cunha L.M., Oliveira J.C., Mahajan P.V., Fonseca S.C. (2017). Modelling the influence of time, temperature and relative humidity conditions on the mass loss rate of fresh oyster mushrooms. J. Food Eng..

[113] Guo Y., Chen X., Gong P., Wang R., Han A., Deng Z., Qi Z., Long H., Wang J., Yao W. (2023). Advances in the Role and Mechanisms of Essential Oils and Plant Extracts as Natural Preservatives to Extend the Postharvest Shelf Life of Edible Mushrooms. Foods.

[114] Dama C.L., Kumar S., Mishra B.K., Shukla K.B., Mathur S., Doshi A. (2010). Antioxidative Enzymatic Profile of Mushrooms Stored at Low Temperature. J. Food Sci. Technol..

[115] bin Harun A. (2017). Post Harvest Control for Maintenance of Quality Mushrooms. Food and Fertilizer Technology Center for the Asian and Pacific Region. https://ap.fftc.org.tw/article/1249.

[116] Gui H., Zhao M., Zhang S., Yin R., Hu C., Fan M., Li L. (2022). Active Antioxidant Packaging from Essential Oils Incorporated Polylactic Acid/Poly(butylene adipate-co-terephthalate)/Thermoplastic Starch for Preserving Straw Mushroom. Foods.

[117] Valderrama F., Ruiz F. (2018). An optimal control approach to steam distillation of essential oils from aromatic plants. Comput. Chem. Eng..

[118] de Souza E.L., Lundgren G.A., de Oliveira K.Á.R., Berger L.R.R., Magnani M. (2019). An Analysis of the Published Literature on the Effects of Edible Coatings Formed by Polysaccharides and Essential Oils on Postharvest Microbial Control and Overall Quality of Fruit. Compr. Rev. Food Sci. Food Saf..

[119] Pandey A.K., Kumar P., Singh P., Tripathi N.N., Bajpai V.K. (2017). Essential Oils: Sources of Antimicrobials and Food Preservatives. Front. Microbiol..

[120] Radünz M., Dos Santos Hackbart H.C., Camargo T.M., Nunes C.F.P., de Barros F.A.P., Dal Magro J., Filho P.J.S., Gandra E.A., Radünz A.L., da Rosa Zavareze E. (2020). Antimicrobial potential of spray drying encapsulated thyme (*Thymus vulgaris*) essential oil on the conservation of hamburger-like meat products. Int. J. Food Microbiol..

[121] Chagas E., Majolo C., Monteiro P., Oliveira M., Gama P., Bizzo H., Chaves F. (2020). Composition of essential oils of Mentha species and their antimicrobial activity against *Aeromonas* spp. J. Essent. Oil Res..

[122] Cai C., Ma R., Duan M., Deng Y., Liu T., Lu D. (2020). Effect of starch film containing thyme essential oil microcapsules on the physicochemical activity of mango. LWT.

[123] Viacava G.E., Ayala-Zavala J.F., González-Aguilar G.A., Ansorena M.R. (2018). Effect of free and microencapsulated thyme essential oil on quality attributes of minimally processed lettuce. Postharvest Biol. Technol..

[124] Hyldgaard M., Mygind T., Meyer R.L. (2012). Essential oils in food preservation: Mode of action, synergies, and interactions with food matrix components. Front. Microbiol..

[125] Perricone M., Arace E., Corbo M.R., Sinigaglia M., Bevilacqua A. (2015). Bioactivity of essential oils: A review on their interaction with food components. Front. Microbiol..

[126] Fiocco D., Fiorentino D., Frabboni L., Benvenuti S., Orlandini G., Pellati F., Gallone A. (2011). Lavender and peppermint essential oils as effective mushroom tyrosinase inhibitors: A basic study. Flavour Fragr. J..

[127] Aly A.A., Mohammed M.K., Maraei R.W., Abdalla A.E., Abouel-Yazeed A.M. (2023). Improving the nutritional quality and bio-ingredients of stored white mushrooms using gamma irradiation and essential oils fumigation. Radiochim. Acta.

[128] Lv F., Liang H., Yuan Q., Li C. (2011). In vitro antimicrobial effects and mechanism of action of selected plant essential oil combinations against four food-related microorganisms. Food Res. Int..

[129] Sánchez-González L., Vargas M., González-Martínez C., Chiralt A., Cháfer M. (2011). Use of Essential Oils in Bioactive Edible Coatings: A Review. Food Eng. Rev..

[130] Jiang T., Luo Z., Ying T. (2015). Fumigation with Essential Oils Improves Sensory Quality and Enhances Antioxidant Ability of Shiitake Mushroom (*Lentinus edodes*). Food Chem..

[131] Chouhan S., Sharma K., Guleria S. (2017). Antimicrobial Activity of Some Essential Oils—Present Status and Future Perspectives. Medicines.

[132] Díaz-Montes E., Castro-Muñoz R. (2021). Edible films and coatings as food-quality preservers: An overview. Foods.

[133] Cavusoglu S., Uzun Y., Yilmaz N., Ercisli S., Eren E., Ekiert H., Elansary H.O., Szopa A. (2021). Maintaining the Quality and Storage Life of Button Mushrooms (*Agaricus bisporus*) with Gum, Agar, Sodium Alginate, Egg White Protein, and Lecithin Coating. J. Fungi.

[134] Louis E., Villalobos-Carvajal R., Reyes-Parra J., Jara-Quijada E., Ruiz C., Andrades P., Gacitúa J., Beldarraín-Iznaga T. (2021). Preservation of mushrooms (*Agaricus bisporus*) by an alginate-based-coating containing a cinnamaldehyde essential oil nanoemulsion. Food Packag. Shelf Life.

[135] Kerch G. (2015). Chitosan films and coatings prevent losses of fresh fruit nutritional quality: A review. Trends Food Sci. Technol..

[136] Pérez-Santaescolástica C., Munekata P.E.S., Feng X., Liu Y., Bastianello Campagnol P.C., Lorenzo J.M. (2022). Active edible coatings and films with Mediterranean herbs to improve food shelf-life. Crit. Rev. Food Sci. Nutr..

[137] Jiang T., Feng L., Zheng X. (2012). Effect of Chitosan Coating Enriched with Thyme Oil on Postharvest Quality and Shelf Life of Shiitake Mushroom (*Lentinus edodes*). J. Agric. Food Chem..

[138] Huang Q., Qian X., Jiang T., Zheng X. (2019). Effect of Chitosan and Guar Gum-Based Composite Edible Coating on Quality of Mushroom (*Lentinus edodes*) during Postharvest Storage. Sci. Hortic..

[139] Shenbagam A., Kumar N., Rahul K., Upadhyay A., Gniewosz M., Kieliszek M. (2023). Characterization of Aloe Vera Gel-Based Edible Coating with Orange Peel Essential Oil and Its Preservation Effects on Button Mushroom (*Agaricus bisporus*). Food Bioprocess Technol..

[140] Gholami R., Ahmadi E., Farris S. (2017). Shelf life extension of white mushrooms (*Agaricus bisporus*) by low temperatures conditioning, modified atmosphere, and nanocomposite packaging material. Food Packag. Shelf Life.

[141] Zhang K., Pu Y.Y., Sun D.W. (2018). Recent advances in quality preservation of postharvest mushrooms (*Agaricus bisporus*): A review. Trends Food Sci. Technol..

[142] Oliveira M., Abadias M., Usall J., Torres R., Teixidó N., Viñas I. (2015). Application of modified atmosphere packaging as a safety approach to fresh-cut fruits and vegetables—A review. Trends Food Sci. Technol..

[143] Joshi K., Warby J., Valverde J., Tiwari B., Cullen P.J., Frias J.M. (2018). Impact of cold chain and product variability on quality attributes of modified atmosphere packed mushrooms (*Agaricus bisporus*) throughout distribution. J. Food Eng..

[144] Commission E. (2009). Commission Regulation (EC) No 450/2009 of 29 May 2009 on active and intelligent materials and articles intended to come into contact with food. Off. J. Eur. Union.

[145] Singh S., Gaikwad K.K., Lee M., Lee Y.S. (2018). Thermally buffered corrugated packaging for preserving the postharvest freshness of mushrooms (*Agaricus bisporus*). J. Food Eng..

[146] Chang C.-K., Cheng K.-C., Hou C.-Y., Wu Y.-S., Hsieh C.-W. (2021). Development of Active Packaging to Extend the Shelf Life of *Agaricus bisporus* by Using Plasma Technology. Polymers.

[147] Shonte T.T., Mulla M.F., Foley L., Pathania S. (2024). Mechanisms of Action and Preservation Effects of Packaging Systems for Mushrooms: Novel Approaches to Preserve Irish Edible Mushrooms. Coatings.

[148] Wrona M., Bentayeb K., Nerín C. (2015). A novel active packaging for extending the shelf-life of fresh mushrooms (*Agaricus bisporus*). Food Control.

[149] Han Lyn F., Maryam Adilah Z.A., Nor-Khaizura M.A.R., Jamilah B., Nur Hanani Z.A. (2020). Application of modified atmosphere and active packaging for oyster mushroom (*Pleurotus ostreatus*). Food Packag. Shelf Life.

[150] Dodero A., Escher A., Bertucci S., Castellano M., Lova P. (2021). Intelligent Packaging for Real-Time Monitoring of Food-Quality: Current and Future Developments. Appl. Sci..

[151] Poyatos-Racionero E., Ros-Lis J.V., Vivancos J.-L., Martínez-Máñez R. (2018). Recent advances on intelligent packaging as tools to reduce food waste. J. Clean. Prod..

[152] Müller P., Schmid M. (2019). Intelligent Packaging in the Food Sector: A Brief Overview. Foods.

[153] Liu D., Zhang C., Pu Y., Chen S., Li H., Zhong Y. (2023). Novel colorimetric films based on polyvinyl alcohol/sodium carboxymethyl cellulose doped with anthocyanins and betacyanins to monitor pork freshness. Food Chem..

[154] Fan S., Mu H., Gao H., Chen H., Wu W., Fang X., Liu R., Niu B. (2023). Preparation of PVA/PLA-based intelligent packaging to indicate the quality of shiitake mushrooms. J. Agric. Food Res..

[155] Liu L., Wu W., Zheng L., Yu J., Sun P., Shao P. (2022). Intelligent packaging films incorporated with anthocyanins-loaded ovalbumin-carboxymethyl cellulose nanocomplexes for food freshness monitoring. Food Chem..

[156] Pérez-Chávez A.M., Alberti M.M., Albertó E. (2022). Evaluation of ligninolytic activity in spent mushroom substrate from four cultivated mushrooms. J. Bioresour. Bioprod..

[157] Hanafi F.H.M., Rezania S., Mat Taib S., Md Din M.F., Yamauchi M., Sakamoto M., Hara H., Park J., Ebrahimi S.S. (2018). Environmentally sustainable applications of agro-based spent mushroom substrate (SMS): An overview. J. Mater. Cycles Waste Manag..

[158] Chang B.-V., Fan S.-N., Tsai Y.-C., Chung Y.-L., Tu P.-X., Yang C.-W. (2018). Removal of emerging contaminants using spent mushroom compost. Sci. Total Environ..

[159] Corral-Bobadilla M., González-Marcos A., Vergara-González E., Alba-Elías F. (2019). Bioremediation of Waste Water to Remove Heavy Metals Using the Spent Mushroom Substrate of *Agaricus bisporus*. Water.

[160] Herrero-Hernández E., Andrades M.S., Villalba Eguren G., Sánchez-Martín M.J., Rodríguez-Cruz M.S., Marín-Benito J.M. (2022). Organic Amendment for the Recovery of Vineyard Soils: Effects of a Single Application on Soil Properties over Two Years. Processes.

[161] Jin Z., Zhang M., Li R., Zhang X., Wang G., Liu X., Qu J., Jin Y. (2020). Spent mushroom substrate combined with alkaline amendment passivates cadmium and improves soil property. Environ. Sci. Pollut. Res. Int..

[162] García-Delgado C., Jiménez-Ayuso N., Frutos I., Gárate A., Eymar E. (2013). Cadmium and lead bioavailability and their effects on polycyclic aromatic hydrocarbons biodegradation by spent mushroom substrate. Environ. Sci. Pollut. Res..

[163] Zied D.C., Sánchez J.E., Noble R., Pardo-Giménez A. (2020). Use of spent mushroom substrate in new mushroom crops to promote the transition towards a circular economy. Agronomy.

[164] Hu W., Di Q., Liang T., Liu J., Zhang J. (2022). Effects of spent mushroom substrate biochar on growth of oyster mushroom (*Pleurotus ostreatus*). Environ. Technol. Innov..

[165] Rajavat A.S., Rai S., Pandiyan K., Kushwaha P., Choudhary P., Kumar M., Chakdar H., Singh A., Karthikeyan N., Bagul S.Y. (2020). Sustainable use of the spent mushroom substrate of *Pleurotus florida* for production of lignocellulolytic enzymes. J. Basic Microbiol..

[166] Grujić M., Dojnov B., Potočnik I., Duduk B., Vujčić Z. (2015). Spent mushroom compost as substrate for the production of industrially important hydrolytic enzymes by fungi *Trichoderma* spp. and *Aspergillus niger* in solid state fermentation. Int. Biodeterior. Biodegrad..

[167] He J., Qiu Y., Ji X., Liu X., Qiu Z., Xu J., Xia J. (2021). A novel strategy for producing cellulase from Trichoderma reesei with ultrasound-assisted fermentation using spent mushroom substrate. Process Biochem..

[168] Branà M., Sergio L., Haidukowski M., Logrieco A., Altomare C. (2020). Degradation of Aflatoxin B1 by a Sustainable Enzymatic Extract from Spent Mushroom Substrate of *Pleurotus eryngii*. Toxins.

[169] Baptista F., Almeida M., Paié-Ribeiro J., Barros A.N., Rodrigues M. (2023). Unlocking the Potential of Spent Mushroom Substrate (SMS) for Enhanced Agricultural Sustainability: From Environmental Benefits to Poultry Nutrition. Life.

[170] Kim J.S., Lee Y.H., Kim Y.I., Ahmadi F., Oh Y.K., Park J.M., Kwak W.S. (2016). Effect of microbial inoculant or molasses on fermentative quality and aerobic stability of sawdust-based spent mushroom substrate. Bioresour. Technol..

[171] Baek Y., Kim M., Reddy K.E., Oh Y., Jung Y., Yeo J., Choi H. (2017). Rumen fermentation and digestibility of spent mushroom (*Pleurotus ostreatus*) substrate inoculated with Lactobacillus brevis for Hanwoo steers. Rev. Colomb. Cienc. Pecu..

[172] Nakatsuka H., Oda M., Hayashi Y., Tamura K. (2016). Effects of fresh spent mushroom substrate of *Pleurotus ostreatus* on soil micromorphology in Brazil. Geoderma.

[173] Lou Z., Sun Y., Zhou X., Baig S.A., Hu B., Xu X. (2017). Composition variability of spent mushroom substrates during continuous cultivation, composting process and their effects on mineral nitrogen transformation in soil. Geoderma.

[174] Lopes A.D., de Melo Santana Gomes S., Schwengber R.P., Carpi M.C.G., Dias-Arieira C.R. (2023). Control of *Meloidogyne javanica* with *Pleurotus djamor* spent mushroom substrate. Chem. Biol. Technol. Agric..

[175] Huang Z., Guan H., Zheng H., Wang M., Xu P., Dong S., Yang Y., Xiao J. (2022). Novel liquid organic fertilizer: A potential way to effectively recycle spent mushroom substrate. J. Clean. Prod..

[176] Van Tam N., Wang C.-H. (2015). Use of Spent Mushroom Substrate and Manure Compost for Honeydew Melon Seedlings. J. Plant Growth Regul..

[177] Najafi B., Faizollahzadeh Ardabili S., Shamshirband S., Chau K. (2019). Spent mushroom compost (SMC) as a source for biogas production in Iran. Eng. Appl. Comput. Fluid Mech..

[178] Grover R., Goel A., Wati L., Raj K. (2015). Ethanol production from spent oyster mushroom substrate. Pollut. Res..

[179] de Almeida Moreira B.R., da Silva Viana R., Magalhães A.C., Caraschi J.C., Zied D.C., Dias E.S., Rinker D.L. (2020). Production of *Pleurotus ostreatus* var. florida on briquettes and recycling its spent substrate as briquettes for fuel grade biosolids. J. Clean. Prod..

[180] Wan-Mohtar W.A.A.Q.I., Halim-Lim S.A., Kamarudin N.Z., Rukayadi Y., Abd Rahim M.H., Jamaludin A.A., Ilham Z. (2020). Fruiting-body-base flour from an Oyster mushroom waste in the development of antioxidative chicken patty. J. Food Sci..

[181] Wan-Mohtar W.A.A.Q.I., Mahmud N., Supramani S., Ahmad R., Zain N.A.M., Hassan N.A.M., Peryasamy J., Halim-Lim S.A. (2018). Fruiting-body-base flour from an oyster mushroom—A waste source of antioxidative flour for developing potential functional cookies and steamed-bun. AIMS Agric. Food.

[182] Wang L., Brennan M.A., Guan W., Liu J., Zhao H., Brennan C.S. (2021). Edible mushrooms dietary fibre and antioxidants: Effects on glycaemic load manipulation and their correlations pre-and post-simulated in vitro digestion. Food Chem..

[183] Harada-Padermo S.D.S., Dias-Faceto L.S., Selani M.M., Alvim I.D., Floh E.I.S., Macedo A.F., Bogusz S., Dias C.T.D.S., Conti-Silva A.C., Vieira T.M.F.S. (2020). Umami Ingredient: Flavor enhancer from shiitake (*Lentinula edodes*) byproducts. Food Res. Int..

[184] Van Ba H., Seo H.-W., Cho S.-H., Kim Y.-S., Kim J.-H., Ham J.-S., Park B.Y., Pil-Nam S. (2016). Antioxidant and anti-foodborne bacteria activities of shiitake by-product extract in fermented sausages. Food Control.

[185] Van Ba H., Seo H.-W., Cho S.-H., Kim Y.-S., Kim J.-H., Ham J.-S., Park B.Y., Pil-Nam S. (2017). Effects of extraction methods of shiitake by-products on their antioxidant and antimicrobial activities in fermented sausages during storage. Food Control.

[186] Banerjee D.K., Das A.K., Banerjee R., Pateiro M., Nanda P.K., Gadekar Y.P., Biswas S., McClements D.J., Lorenzo J.M. (2020). Application of Enoki Mushroom (*Flammulina Velutipes*) Stem Wastes as Functional Ingredients in Goat Meat Nuggets. Foods.

[187] Gonzalez A., Cruz M., Losoya C., Nobre C., Loredo A., Rodríguez-Jasso R.M., Contreras J.C., Belmares R. (2020). Edible Mushrooms as a Novel Protein Source for Functional Foods. Food Funct..

[188] Rangel-Vargas E., Rodriguez J.A., Domínguez R., Lorenzo J.M., Sosa M.E., Andrés S.C., Rosmini M., Pérez-Alvarez J.A., Teixeira A., Santos E.M. (2021). Edible Mushrooms as a Natural Source of Food Ingredient/Additive Replacer. Foods.

